# Conservation agriculture and climate resilience^☆^

**DOI:** 10.1016/j.jeem.2018.11.008

**Published:** 2019-01

**Authors:** Jeffrey D. Michler, Kathy Baylis, Mary Arends-Kuenning, Kizito Mazvimavi

**Affiliations:** a Dept. of Agricultural and Resource Economics, University of Arizona, Tucson, USA; b Dept. of Agricultural and Consumer Economics, University of Illinois, Urbana, USA; c International Crops Research Institute for the Semi-Arid Tropics, Bulawayo, Zimbabwe

**Keywords:** Conservation farming, Technology adoption, Climate smart agriculture, Weather risk, Zimbabwe

## Abstract

Agricultural productivity growth is vital for economic and food security outcomes which are threatened by climate change. In response, governments and development agencies are encouraging the adoption of ‘climate-smart’ agricultural technologies, such as conservation agriculture (CA). However, there is little rigorous evidence that demonstrates the effect of CA on production or climate resilience, and what evidence exists is hampered by selection bias. Using panel data from Zimbabwe, we test how CA performs during extreme rainfall events - both shortfalls and surpluses. We control for the endogenous adoption decision and find that use of CA in years of average rainfall results in no yield gains, and in some cases yield loses. However, CA is effective in mitigating the negative impacts of deviations in rainfall. We conclude that the lower yields during normal rainfall seasons may be a proximate factor in low uptake of CA. Policy should focus promotion of CA on these climate resilience benefits.

## 1 Introduction

Smallholder agriculture is not just a source of food but a driver of economic development, particularly for the 75 percent of the world's poor who live in rural areas. However, agricultural production is straining natural resources, suggesting that productivity improvements are required to feed a growing population (FAO et al., 2017). Agriculture and food security are further threatened by climate change, particularly in Sub-Saharan Africa, and particularly for smallholder farmers (Morton, 2007; Schlenker and Lobell, 2010; Wheeler and von Braun, 2013). Climate change has already substantially reduced production in many parts of the world (Lobell et al., 2011). In response, governments and development agencies are encouraging the adoption of ‘climate smart’ agricultural technologies, including conservation agriculture, with the goal of bolstering productivity, enhancing resilience to weather shocks, and reducing negative externalities (FAO, 2013; Lipper et al., 2014).

Conservation agriculture (CA) is based on three practices promoted as a means for sustainable agricultural intensification: minimum tillage, mulching with crop residue, and crop rotation. The goal of these practices is to increase yields through improvements in soil fertility, and reduce risk to yields from rainfall shocks (Brouder and Gomez-Macpherson, 2014).^1^ In addition to the stated private benefits of increased productivity and enhanced yield resilience, proponents claim that CA has positive environmental externalities by increasing soil organic carbon (Smith et al., 1998; Lal and Stewart, 2010).^2^ Yet adoption rates of CA have been low, and disadoption rates are high, limiting the scope of any positive externalities and raising the question of whether farmers receive the promised private benefits (Pedzisa et al., 2015a). The little evidence that exists regarding CA's ‘climate smart’ properties mainly relies on agronomic analysis of field station trials, missing potential effects of farmer behavior. As Andersson and D'Souza (2014) and Pannell et al. (2014) observe, the few studies that do use observational data often fail to account for selection bias or fully control for all potential sources of endogeneity.

In this paper, we compare CA's effect on overall yield to its potential in reducing yield losses due to deviations in average rainfall, controlling for potential selection bias. While CA is hypothesized to be ‘climate smart,’ increased resilience during periods of rainfall stress may come at the cost of yields during regular growing conditions. We follow Di Falco and Chavas (2008) by defining resilience in terms of practices that limit productivity loss when challenged by climatic events. We use four years of plot-level panel data covering 4, 171 plots from 729 households across Zimbabwe to estimate how yields respond during periods of high and low rainfall shocks, compared to the norms households experience over time. The data include plot-level inputs and output for five different crops: maize, sorghum, millet, groundnut, and cowpea. Unlike earlier work that estimates CA's effect on a single crop, this rich set of production data allows us to examine how the impacts of CA vary in the multi-cropping system common across Sub-Saharan Africa.

Causal identification of CA's impact on yields is complicated by non-random adoption of the technology.^3^ We identify two potential sources of endogeneity that might bias our results. First is the presence of unobserved household heterogeneity that influences both adoption and yields, which we control for with household fixed effects. Second is the possible presence of unobserved time-varying shocks that might affect a household's access to and use of CA while being correlated with yields. To instrument for household adoption, we use data on CA promotion through the distribution of subsidized inputs as part of the Zimbabwe Protracted Relief Programme (PRP). The PRP was a four year, *£*28 million project aimed at providing short-term nutritional, economic, and agricultural interventions to one-third of all smallholder households in the country, about 1.7 million people (Jennings et al., 2013). In addition to these two primary concerns, we conduct a number of robustness checks, including controlling for plot-level unobservables, changes to the functional form, testing for the impact of rainfall event outliers, and changing our definition of rainfall shock.

We find that adoption of CA in years of average rainfall results in no yield gains, and in some cases yield loses, compared to conventional practices. Where CA is effective is in mitigating the negative impacts of deviations in rainfall. The magnitude of these resilience effects vary by crop, and whether rainfall is in shortfall or surplus. We then use the estimated coefficients on the CA terms to predict the returns to CA at various values of rainfall. A one standard deviation decrease or increase in rainfall is required before the returns to CA become positive. Our results, using observational data, support a recent meta-analysis of agronomic field experiments which found that, on average, CA reduces yields but can enhance yields in dry climates (Pittelkow et al., 2015).

Pannell et al. (2014) discuss several limitations with the state of economic research on CA. A key limitation is a lack of clarity regarding what qualifies as CA adoption. Our survey data allow us to partially address this concern. While the data cover four cropping seasons, only in the final two seasons were households asked about which specific practices they implemented on plots that they identified as cultivating with CA. Among households that used CA in the last two years of the survey, 97 percent of them used planting basins to achieve minimum soil disturbance, 27 percent mulched with crop residue, and 36 percent practiced crop rotation. In total, 22 percent of self-identified CA adopters engaged in at least two practices while only five percent of CA adopters actually engaged in all three practices. Therefore, in our context, we use a practical definition of CA as, at the very least, minimum tillage, the original and central principle of CA. We then define traditional cultivation practices as everything other than self-identified CA adoption, which for the vast majority of plots amounts to conventional tillage practices. While our pragmatic approach is not ideal, it is in line with previous literature at the farm level and with meta-analyses of agronomic field experiment data.

This paper makes three contributions to the literature. First, by interacting the instrumented measure of CA adoption with deviations in rainfall we directly test whether CA can mitigate yield loss due to adverse weather events. The only evidence of the resilience of yields under CA comes from station and on-farm field trial data or from observational studies that fails to adequately control for both time-variant and time-invariant sources of endogeneity. In this literature, researchers acknowledge that selection bias is a problem because farmers choose the technology based on its expected benefits, which are heterogeneous. Much of the literature relies on cross-sectional data and techniques that involve estimating selection correction terms (Kassie et al., 2009; Teklewold et al., 2013a, b; Kassie et al., 2015a, b; Abdulai, 2016; Manda et al., 2016). Like any other IV, selection models rely on exclusion restrictions for proper identification of time-variant endogenous choice variables. However, reliance on cross-sectional data means that one cannot easily control for endogeneity coming from time-invariant sources. A second, related, set of literature relies on panel data to control for time-invariant heterogeneity (Arslan et al., 2014, 2015; Ndlovu et al., 2014; Pedzisa et al., 2015a, b). This literature, though, fails to control for time-variant factors causing endogeneity in the adoption decision. We use realizations of on-farm yields over four years and control for both sources of endogeneity. Ours is one of the first papers to test the ‘climate smart’ properties of CA as they are experienced by farm households.

Second, we expand the analysis of CA by encompassing a variety of crops. Most previous work focuses on the impact of CA on yields for a single crop, often maize (Teklewold et al., 2013a, b; Brouder and Gomez-Macpherson, 2014; Kassie et al., 2015a, b; Manda et al., 2016). But, farmers in Sub-Saharan Africa frequently grow multiple crops in a single season, meaning that decisions regarding cultivation method and input use are made at the farm level, not the crop level. To examine the farm-wide impacts, we adopt a flexible yield function that allows coefficients on inputs to vary across crops. This approach accommodates heterogeneity in the input-response curves by allowing slopes and intercepts to vary across crops without forcing us to split the random sample based on non-random criteria. Our results provide new insight on the use and impact of CA in a multi-cropping environment.

Third, we provide suggestive evidence on the reason for the low adoption rate of CA among farmers in Sub-Saharan Africa. Given early evidence on the positive correlation between CA and yields (Mazvimavi and Twomlow, 2009), and its promotion by research centers, donor agencies, and governments (Andersson and D'Souza, 2014), the slow uptake of CA has presented the adoption literature with an empirical puzzle. Once we control for endogeneity in the adoption decision, CA frequently has a negative impact on yields during periods of average rainfall. We conclude that the lack of yield gains during average rainfall seasons may be a limiting factor in the uptake of CA, and by extension limits any positive environmental externalities CA may provide. Focusing on CA's potential as a ‘climate smart’ agricultural technology by advertising the resilience benefits of CA marks a path forward for the promotion of CA in regions facing climate extremes.

## 2 Theoretical framework

We begin by defining two stochastic Cobb-Douglas yield functions along the lines of Just and Pope (1978), Barrett et al. (2004), and Suri (2011):

(1) $$\begin{array}{cc} Y_{kit}^{CA\;} & =e^{\beta_{kt}^{CA\;}}\left(\overset{J}{\underset{j=1}{\prod }}X_{jkit}^{\gamma_{jk}^{CA\;}}\right)e^{u_{kit}^{CA\;}}, \end{array}$$

(2) $$\begin{array}{cc} Y_{kit}^{TC\;} & =e^{\beta_{kt}^{TC\;}}\left(\overset{J}{\underset{j=1}{\prod }}X_{jkit}^{\gamma_{jk}^{TC\;}}\right)e^{u_{kit}^{TC\;}}, \end{array}$$

where *Y*_*kit*_ is yield for crop *k* cultivated by household *i* at time *t* and *X*_*jkit*_ is a set of *j* measured inputs, including a crop-specific intercept, used on the *k*th crop by household *i* at time *t*. We allow the yield functions for conservation agriculture (CA) and traditional cultivation (TC) to have different parameters for inputs ($\gamma_{j}^{CA\;}$ and $\gamma_{j}^{TC\;}$), although the same set of potential inputs are used in both cultivation methods. The disturbance terms ($u_{kit}^{CA\;}$ and $u_{kit}^{TC\;}$) are sector-specific errors composed of time-invariant farm and household characteristics and time-variant production shocks.

Taking logs of Eqs. (1), (2) gives us:

(3) $$\begin{array}{cc} y_{kit}^{CA\;} & =\beta_{kt}^{CA\;}+x_{jkit}^{\prime }\gamma_{jk}^{CA\;}+u_{kit}^{CA\;}, \end{array}$$

(4) $$\begin{array}{cc} y_{kit}^{TC\;} & =\beta_{kt}^{TC\;}+x_{jkit}^{\prime }\gamma_{jk}^{TC\;}+u_{kit}^{TC\;}, \end{array}$$

where we can decompose the disturbance terms into:

(5) $$\begin{array}{cc} u_{kit}^{CA\;} & =\theta_{ki}^{CA\;}+\xi_{kit}^{CA\;}, \end{array}$$

(6) $$\begin{array}{cc} u_{kit}^{TC\;} & =\theta_{ki}^{TC\;}+\xi_{kit}^{TC\;}. \end{array}$$

The *θ*_*ki*_'s are crop-level productivity terms based on factors not chosen but known by the household. The transitory errors ($\xi_{kit}^{CA\;}$ and $\xi_{kit}^{TC\;}$) are assumed to be uncorrelated with each other, uncorrelated with the *X*_*jkit*_'s, unknown by households, and have a zero mean and finite variance.

We can define the relative productivity of a plot cultivated using CA compared to traditional methods as $\left(\theta_{ki}^{CA\;}-\theta_{ki}^{TC\;}\right)$, which relies on the decomposition of $\theta_{ki}^{CA\;}$ and $\theta_{ki}^{TC\;}$ into:

(7) $$\begin{array}{cc} \theta_{ki}^{CA\;} & =b_{k}^{CA\;}\left[\overset{T}{\underset{t=1}{\sum }}\left({R\;}_{kit}^{CA\;}-{R\;}_{kit}^{TC\;}\right)\right]+\tau_{i}, \end{array}$$

(8) $$\begin{array}{cc} \theta_{ki}^{TC\;} & =b_{k}^{TC\;}\left[\overset{T}{\underset{t=1}{\sum }}\left({R\;}_{kit}^{CA\;}-{R\;}_{kit}^{TC\;}\right)\right]+\tau_{i}. \end{array}$$

The *b*_*k*_'s are a measure of each crop's relative advantage under a given cultivation method and for a certain level of rainfall, R. The *τ*_*i*_ term is the household's absolute advantage in cultivating crops, which does not vary by crop type or cultivation method and is constant over time.

We can re-define this comparative advantage gain as:

(9) $$\theta_{ki}\equiv b_{k}^{TC\;}\left[\overset{T}{\underset{t=1}{\sum }}\left({R\;}_{kit}^{CA\;}-{R\;}_{kit}^{TC\;}\right)\right]$$

and define a new parameter, $\phi_{k}\equiv b_{k}^{CA\;}/b_{k}^{TC\;}-1$. This allows us to re-write Eqs. (7), (8) as:

(10) $$\begin{array}{cc} \theta_{ki}^{CA\;} & =\left(\phi +1\right)\theta_{ki}+\tau_{i}, \end{array}$$

(11) $$\begin{array}{cc} \theta_{ki}^{TC\;} & =\theta_{ki}+\tau_{i}. \end{array}$$

Substituting the decomposed error terms back into Eqs. (3), (4) results in:

(12) $$\begin{array}{cc} y_{kit}^{CA\;} & =\beta_{kt}^{CA\;}+x_{jkit}^{\prime }\gamma_{jk}^{CA\;}+\left(\phi +1\right)\theta_{ki}+\tau_{i}+\xi_{kit}^{CA\;}, \end{array}$$

(13) $$\begin{array}{cc} y_{kit}^{TC\;} & =\beta_{kt}^{TC\;}+x_{jkit}^{\prime }\gamma_{jk}^{TC\;}+\theta_{ki}+\tau_{i}+\xi_{kit}^{TC\;}. \end{array}$$

Using a generalized yield function of the form

(14) $$y_{kit}=h_{kit}y_{kit}^{CA\;}+\left(1-h_{kit}\right)y_{kit}^{TC\;},$$

we can substitute Eqs. (12), (13) into Eq. (14) to get:

(15) $$y_{kit}=\beta_{kt}^{TC\;}+\theta_{ki}+\left(\beta_{kt}^{CA\;}-\beta_{kt}^{TC\;}\right)h_{kit}+x_{jkit}^{\prime }\gamma_{jk}^{TC\;}+\phi_{k}\theta_{ki}h_{kit}+x_{jkit}^{\prime }\left(\gamma_{jk}^{CA\;}-\gamma_{jk}^{TC\;}\right)h_{kit}+\tau_{i}+\varepsilon_{kit}.$$

Here *h*_*kit*_ is an indicator of whether or not crop *k* was cultivated by household *i* at time *t* using CA. The *θ*_*ki*_ term is the impact of rainfall, or rainfall shocks, on crop *k* grown by household *i*. We are primarily interested in the coefficient *ϕ*_*k*_ on the composite term *θ*_*ki*_*h*_*kit*_, which measures the impact of rainfall on yield given a household was using CA.

The expected gain from using CA is therefore:

(16) $$B_{kit}=y_{kit}^{CA\;}-y_{kit}^{TC\;}=\phi_{k}\theta_{ki}+\left(\beta_{kt}^{CA\;}-\beta_{kt}^{TC\;}\right)+x_{jkit}^{\prime }\left(\gamma_{jk}^{CA\;}-\gamma_{jk}^{TC\;}\right).$$

Neither the household fixed effect term nor the transitory error term appear in Eq. (16). This is because *τ*_*i*_ is restricted to impact yields identically regardless of cultivation method and because of the assumptions placed on $\xi_{kit}^{CA\;}$ and $\xi_{kit}^{TC\;}$. These logarithmic representations of the yield function map back to a generalized Cobb-Douglas yield function in which returns are heterogeneous based on crop type and cultivation method. While it is recognized that the Cobb-Douglas is not a flexible functional form, in studies of developing country agriculture it is often still preferred to the translog due to data limitations, the simplicity of the production technology, and the frequent issue of multicollinearity in estimation (Michler and Shively, 2015).

Our primary empirical specification is contained in Eq. (15). To implement the estimation procedure we make two simplifying assumptions. First, we allow $\beta_{kt}^{CA\;}-\beta_{kt}^{TC\;}=\beta_{kt}$ and assume *β*_*kt*_ = *β*_*k*_ *∀ t*. This amounts to assuming that, holding rainfall constant, productivity gains from adoption of CA vary by crop but not over time. While there is evidence from experimental plots that CA improves productivity over time, this typically requires ten to fifteen years (Giller et al., 2009). Given that our panel only covers four years, we do not expect productivity gains to vary over this short of a time frame. Second, we assume $\gamma_{jk}^{CA\;}=\gamma_{jk}^{TC\;}=\gamma_{jk}$. This allows the yield response curves to vary by input and by crop but not by cultivation method. We address this second assumption in greater detail in Section 4.1.

Finally, a potential issue with identifying the impact of CA adoption on yields is that the decision to adopt, *h*_*kit*_, may be correlated with transitory shocks contained in *ε*_*kit*_. In our empirical implementation we instrument for the likely endogeneity of the adoption term using an approach outlined by Wooldridge (2003). We discuss this approach more fully in Section 4.2.

## 3 Data

This study uses four years of panel data on smallholder farming practices in Zimbabwe collected by the International Crops Research Institute for the Semi-Arid Tropics (ICRISAT). The data cover 783 households in 45 wards from 2007-2011.^4^ The wards come from 20 different districts which were purposefully selected to provide coverage of high rainfall, medium rainfall, semi-arid, and arid regions. Thus the survey can be considered nationally representative of smallholder agriculture in Zimbabwe. For our analysis we use an unbalanced panel consisting of a subset of 728 randomly selected households. The 55 excluded households come from the 2007 round, which we drop completely, because in that year the survey only targeted households who had received NGO support as part of the Zimbabwe PRP.

### 3.1 Household data

Our data provide us with detailed information on the cultivation of five crops on 4, 171 unique plots (see Table 1). Maize, the staple grain of Zimbabwe, is the most commonly cultivated crop. Just over half of all observations are maize, and 98 percent of households grow maize on at least one plot in every year. The next most common crop, in terms of observations, is groundnut, which is often grown in rotation with maize. The third most common crop is sorghum, which is frequently grown as an alternative to maize in the semi-arid regions of Zimbabwe. As an alternative to groundnut and sorghum, households will often grow cowpea or pearl millet, respectively. Both of these crops are much less common and combine to account for only 15 percent of observations.

**Table 1:** Descriptive statistics by crop.

	Maize	Sorghum	Millet	Groundnut	Cowpea	Total
Yield (kg/ha)	1217 (1366)	827.7 (1137)	641.0 (946.7)	1065 (1251)	662.4 (1137)	1040 (1284)
CA (= 1)	0.350 (0.477)	0.263 (0.440)	0.137 (0.345)	0.193 (0.395)	0.313 (0.464)	0.290 (0.454)
Basal applied fertilizer (kg)	13.41 (30.39)	2.281 (10.35)	0.923 (5.952)	1.429 (7.653)	3.457 (12.19)	7.716 (23.21)
Top applied fertilizer (kg)	17.58 (32.56)	3.366 (10.69)	1.636 (8.786)	1.843 (9.264)	4.112 (13.11)	10.16 (25.32)
Seed (kg)	8.143 (9.169)	4.896 (6.460)	5.974 (11.40)	10.96 (14.80)	3.099 (4.047)	7.543 (10.20)
Area (m^2^)	3466 (3782)	3225 (3697)	3978 (4138)	2060 (2100)	1553 (1894)	3035 (3486)
Rainfall shock	0.469 (0.274)	0.482 (0.267)	0.551 (0.301)	0.469 (0.273)	0.463 (0.258)	0.476 (0.274)
Number of HH in ward with NGO support	20.62 (13.09)	22.01 (15.51)	24.75 (17.62)	21.77 (14.12)	23.29 (15.15)	21.56 (14.25)
number of observations	3827	1264	488	1397	667	7643
number of plots	2643	1007	405	1220	615	4171
number of households	715	415	177	598	388	728
number of wards	45	41	26	45	43	45

*Note*: The first five columns of the table display means of the data by crop with standard deviations in parenthesis. The final column displays means and standard deviations for the pooled data. Inputs are measured at the plot-level.

**Fig. 1:**
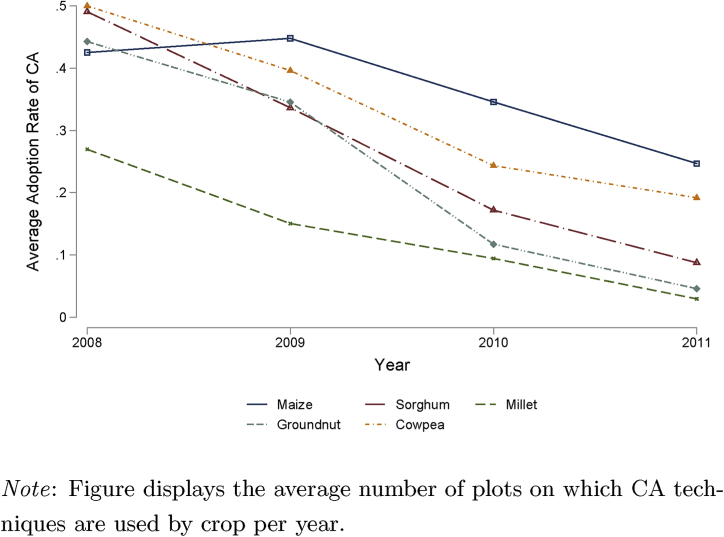
Average annual level of CA adoption by crop.

Examining the production data by year reveals a high degree of annual variability (see Table 2).^5^ Yields in 2009 and 2010 were about 30 percent higher than yields in 2008 or in 2011, despite much higher levels of fertilizer and seed use in the low yield years. CA practices vary both by crop and over time (see Fig. 1). In 2008, adoption of CA was at over 40 percent for those cultivating maize, sorghum, groundnut, and cowpea and it was about 30 percent for those cultivating millet. Since then, adoption of CA has declined for all crops so that the average adoption rate is now only 17 percent, although use of CA for maize cultivation remains relatively high at 25 percent. Previous literature has hypothesized that this abandonment of CA by households in Zimbabwe is due to the withdrawal of NGO input support as the PRP was scaled down (Ndlovu et al., 2014; Pedzisa et al., 2015b). We make use of this insight to help identify CA adoption.

**Table 2:** Descriptive statistics by year.

	2008	2009	2010	2011
Yield (kg/ha)	760.6 (1210)	1278 (1355)	1151 (1415)	936.0 (1094)
CA (= 1)	0.435 (0.496)	0.385 (0.487)	0.247 (0.431)	0.170 (0.376)
Basal applied fertilizer (kg)	7.044 (20.92)	3.898 (14.73)	4.335 (15.26)	14.00 (32.48)
Top applied fertilizer (kg)	8.679 (20.16)	6.776 (16.82)	7.331 (20.15)	16.14 (34.80)
Seed (kg)	7.961 (10.02)	6.399 (7.737)	7.133 (12.29)	8.499 (9.729)
Area planted (m^2^)	3473 (4172)	2870 (3896)	2891 (3348)	3015 (2714)
Rainfall shock	0.682 (0.289)	0.487 (0.267)	0.351 (0.225)	0.453 (0.231)
Number of HH in ward with NGO support	27.87 (19.20)	18.00 (10.38)	21.63 (13.34)	20.21 (12.58)
number of observations	1452	1732	2116	2343
number of plots	1403	1677	2015	2312
number of households	388	401	432	584
number of wards	29	30	31	43

*Note*: Columns in the table display means of the data by year with standard deviations in parenthesis. Inputs are measured at the plot-level.

### 3.2 Rainfall data

To calculate rainfall shocks we use satellite imagery from the Climate Hazards Group InfraRed Precipitation with Station (CHIRPS) data. CHIRPS is a thirty year, quasi-global rainfall dataset that spans 50°S-50°N, with all longitudes and incorporates 0.05° resolution satellite imagery with in-situ station data to create a gridded rainfall time series (Funk et al., 2015). The dataset provides daily rainfall measurements from 1981 up to the current year. We overlay ward boundaries on the 0.05° grid cells and take the average rainfall for the day within the ward. We then aggregate the ward level daily rainfall data to the seasonal level.^6^

**Fig. 2:**
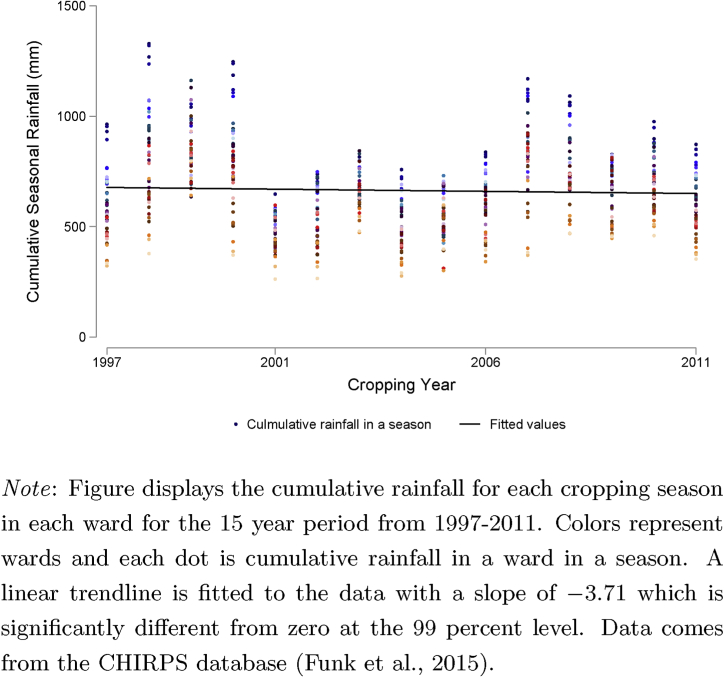
Historic seasonal rainfall by ward.

Fig. 2 shows historic seasonal rainfall distribution by ward over the 15 year period 1997–2011. We observe large seasonal fluctuations as well as longer term cycles in rainfall patterns. The four-year period 1997–2000 saw relatively high levels of rainfall. This was followed by a six-year period in which rainfall was relatively scarce. Since 2007, and throughout the survey period, rainfall was again relatively abundant. It is important to note that only one year in the survey period, 2011, experienced below average rainfall. To some extent, our results will be driven by the yield outcomes from the single drought year in our study.^7^

We follow Ward and Shively (2015) in measuring rainfall shocks as normalized deviations in a single season's rainfall from expected seasonal rainfall over the 15 year period 1997–2011:

(17) $${R\;}_{wt}=\left|\frac{r_{wt}-{\bar{r}}_{w}}{\sigma_{r_{w}}}\right|.$$

Here shocks are calculated for each ward *w* in year *t* where *r*_*wt*_ is the observed amount of rainfall for the season, ${\bar{r}}_{w}$ is the average seasonal rainfall for the ward over the period, and $\sigma_{r_{w}}$ is the standard deviation of rainfall during the same period. Our rainfall shock variable treats too little rain as having the same effect as too much rain. While this may not appear to be meaningful in an agronomic sense, proponents of CA claim that CA will have a positive impact on yields in both abnormally wet and abnormally dry conditions (Thierfelder and Wall, 2009).

We also calculate a measure of rainfall shortage as:

(18) $${\underline{R\;}}_{wt}=\left\{\begin{array}{cc} \left|\frac{r_{wt}-{\bar{r}}_{w}}{\sigma_{r_{w}}}\right|\; & \text{if}\;r_{wt}<{\bar{r}}_{w}\\ 0\; & \text{otherwise} \end{array}\right),$$

as well as a measure of rainfall surplus:

(19) $${\bar{R\;}}_{wt}=\left\{\begin{array}{cc} \left|\frac{r_{wt}-{\bar{r}}_{w}}{\sigma_{r_{w}}}\right|\; & \text{if}\;r_{wt}>{\bar{r}}_{w}\\ 0\; & \text{otherwise} \end{array}\right).$$

These measures help clarify if CA's impact on yield resilience is primarily due to mitigating loss from drought or from excess rainfall.

One potential concern with our rainfall terms is that they are measures of annual deviations in meteorological data which are designed to proxy for agricultural drought or overabundance of rainfall. In Zimbabwe, agricultural drought can take the form of late onset of rains or mid-season dry spells. A further complication is that rainfall in Zimbabwe is often of high intensity but low duration and frequency, creating high runoff and little soil permeation. To test for this, we examine the impact of both seasonal and monthly deviations in rainfall on crop output. We find little empirical evidence that late onset of rainfall or mid-season dry spells reduce yields. Results from these regressions, as well as a more detailed discussion of historical rainfall patterns, are presented in Online Appendix A. Based on these results we conclude that our use of seasonal deviations is a strong proxy for rainfall-induced stresses to agricultural production.

## 4 Identification strategy

Our analysis is based on observational data from a setting in which households were randomly selected into the survey but adoption of CA was not random. Thus, care must be taken in understanding what constitutes the relevant comparable group for hypothesis testing. Additionally, identification of the impact of CA on yields is confounded by two potential sources of endogeneity. First, time-invariant unobserved household-level characteristics might influence both CA adoption and yields. Second, time-variant unobserved shocks captured in *ε*_*kit*_ may simultaneously affect the adoption decision and crop yields.

### 4.1 Establishing the relevant comparison group

While we want to compare adopters to non-adopters, if CA requires different levels of inputs, or if yields respond in different ways to inputs under CA compared to traditional cultivation (TC), we cannot simply compare across the two groups, irrespective of any selection issues.

In Table 3 we explore the differences in outputs and input use by crop across cultivation practices.^8^ Mean yields for all crops are significantly higher under CA than under TC methods. One obvious potential reason why CA is often associated with higher yields is that households increase the intensity of agricultural input application under CA. Compared to TC methods, CA is associated with significantly higher levels of both basal and top applied fertilizer. Conversely, more seed and land is used in TC compared to CA for most crops. The different rates of input use between CA and TC means that the two cultivation techniques are not directly comparable without taking into consideration measured inputs. Because we observe the quantity of fertilizer, seed applied, and the size of each plot, we can directly control for these differences in our econometric analysis.

**Table 3:** Descriptive statistics by crop and cultivation method.

	Maize			Sorghum			Millet			Groundnut			Cowpea		
	TC	CA	MW-test	TC	CA	MW-test	TC	CA	MW-test	TC	CA	MW-test	TC	CA	MW-test
Yield(kg/ha)	932.6 (1127)	1745 (1595)	^∗∗∗^	736.8 (1073)	1082 (1267)	^∗∗∗^	627.7 (979.8)	724.1 (703.2)	^∗∗^	978.4 (1117)	1428 (1665)	^∗∗∗^	626.5 (1153)	740.9 (1101)	^∗∗∗^
Basal applied fertilizer (kg)	11.46 (31.01)	17.03 (28.86)	^∗∗∗^	1.149 (8.582)	5.457 (13.73)	^∗∗∗^	0.705 (6.151)	2.291 (4.284)	^∗∗∗^	0.547 (4.424)	5.106 (14.32)	^∗∗∗^	1.417 (7.236)	7.925 (18.22)	^∗∗∗^
Top applied fertilizer (kg)	15.11 (32.24)	22.18 (32.65)	^∗∗∗^	0.996 (4.346)	10.01 (17.96)	^∗∗∗^	0.724 (5.712)	7.365 (17.98)	^∗∗∗^	0.591 (4.137)	7.065 (18.43)	^∗∗∗^	1.298 (6.539)	10.27 (20.01)	^∗∗∗^
Seed planted (kg)	9.287 (10.61)	6.021 (4.931)	^∗∗∗^	5.206 (5.776)	4.023 (8.023)	^∗∗∗^	6.477 (12.15)	2.812 (2.750)	^∗∗∗^	12.38 (16.01)	5.027 (4.570)	^∗∗∗^	2.957 (4.432)	3.408 (3.023)	^∗∗∗^
Area planted (m^2^)	3988 (4299)	2496 (2257)	^∗∗∗^	3809 (4038)	1582 (1619)	^∗∗∗^	4465 (4227)	915.1 (1249)	^∗∗∗^	2260 (2191)	1223 (1386)	^∗∗∗^	1782 (2119)	1051 (1116)	^∗∗∗^
Rainfall shock	0.465 (0.273)	0.473 (0.275)		0.473 (0.262)	0.503 (0.275)		0.547 (0.309)	0.568 (0.241)		0.455 (0.266)	0.527 (0.293)	^∗∗∗^	0.466 (0.260)	0.453 (0.252)	
Number of HH in ward with NGO support	19.50 (12.85)	22.68 (13.26)	^∗∗∗^	21.23 (14.69)	24.18 (17.42)	^∗∗^	23.26 (17.01)	34.13 (18.52)	^∗∗∗^	20.41 (13.32)	27.40 (15.88)	^∗∗∗^	21.54 (14.31)	27.11 (16.21)	^∗∗∗^
number of observations	2486		421	67		1127	270		458	209					
number of plots	2001	966		792	277		357	58		1015	254		437	198	
number of households	670	537		369	206		168	44		562	188		306	153	
number of wards	45	44		40	30		26	10		45	24		43	30	

*Note*: Columns in the table display means of the data by crop with standard deviations in parenthesis. Columns headed TC are output and inputs used under traditional cultivation practices while columns headed CA are output and inputs used under conservation agriculture. The final column for each crop presents the results of Mann-Whitney two-sample tests for differences in distribution. Results are similar if a Kolmogorov-Smirnov test is used. Significance of MW-tests are reported as ∗p < 0.1; ∗∗p < 0.05; ∗∗∗p < 0.01.

**Fig. 3:**
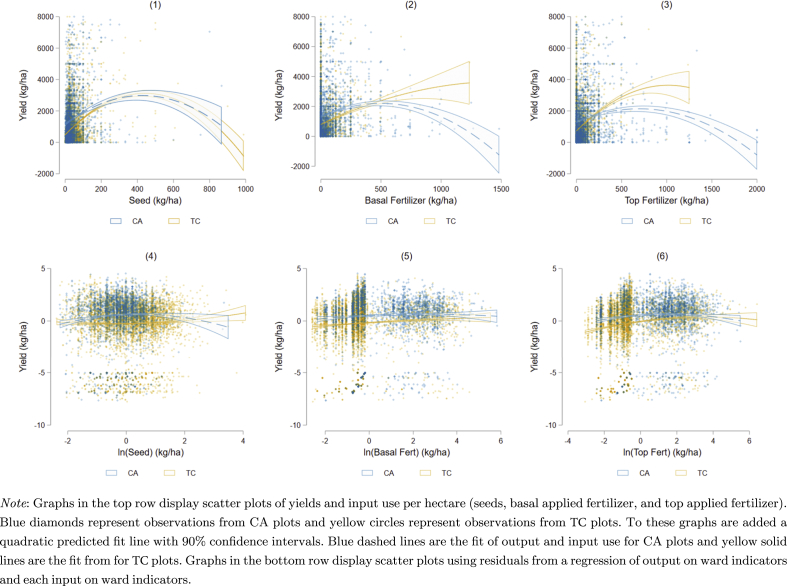
Yield response to inputs by CA adoption.

Directly controlling for input use in our regression framework only allows us to compare CA adopters to non-CA adopters (ignoring issues of selection), if our assumption that yield response curves do not vary by cultivation method holds. We can verify this by graphing the correlation between yields and inputs. In the top row of Fig. 3 we draw scatter plots of yields and seeding rates in panel (1), yields and basal fertilizer application rates in panel (2), and yields and top fertilizer application rates in panel (3). Blue diamonds represent observations from CA plots and yellow circles represent observations from TC plots. We then fit a quadratic function to the data along with a 90 percent confidence interval band. Panels (1)–(3) imply that CA does not affect the responsiveness of yields to seed but may affect the responsiveness of yields to both basal and top applied fertilizer. Thus, we cannot compare yields of CA plots to TC plots, even when explicitly controlling for the amount of measured inputs applied to the plot.

Our data were collected during the implementation of the Zimbabwe Protracted Relief Program (PRP). The PRP simultaneously promoted CA and provided access to subsidized fertilizer and seed. While the subsidy program provided nearly universal coverage at the district level, within districts program support varied from ward-to-ward. Thus, a household's access to subsidized inputs varies depending on which ward a household lives in. To examine if the difference in yield response to inputs across cultivation method is a result of access to inputs, and not a result of CA itself, we estimate four different regressions: log of yield on ward indicators, log of seed on ward indicators, log of basal fertilizer on ward indicators, and log of top fertilizer on ward indicators. We then plot the residuals from these different regressions and again fit a quadratic function to the data (see panels (4)–(6) in Fig. 3). The result allows us to compare yield response by cultivation method within a ward, and thus control for different rates of access to inputs across wards. Comparing panels in the top of Fig. 3 to panels in the bottom, much of the apparent differences in yield response to inputs is actually a result of ward effects. While differences in yield response do not completely disappear, the differences that remain are small compared to differences coming from variation in access to inputs. We conclude that after controlling for ward, our assumption that yield response curves do not vary by cultivation method appears to hold.^9^

In our regression analysis we control for differences in access to inputs resulting from ward location using household fixed effects. Additionally, household fixed effects remove any other unobserved time-invariant household effects, such as skill at farming, wealth, or risk aversion, that may be correlated with both the *τ*_*i*_ term, the decision to adopt, and yields in Eq. (15). Thus, we are estimating the yield response to CA within a household during periods of high and low rainfall shocks, compared to the norms a household experiences over time.

Within a household, farmers may choose to apply CA techniques to some plots and not others based on plot-level unobservables. To test whether this is a valid concern, we compare output and input use across two different “treatment” and “control” groups. In the first, we compare plots on which CA was used in all four rounds of the data (always adopters) with plots that were cultivated using CA in 2008 but which reverted to TC practices in subsequent years (future disadopters). In the second, we compare plots that never experienced CA (never adopters) with plots that were cultivated using TC in 2008 but which adopted CA in subsequent years (future adopters). The intuition behind comparing these plot-level adoption types is that if differences in output and input use exist only after adoption histories diverge, then plot-level unobservables are not a concern. Examining Table 4, we see that in 2008 output and input use levels tend to be statistically indistinguishable from each other.^10^ The two exceptions are the seeding rate between always adopters and future disadopters, and the rate of application of top fertilizer between never adopters and future adopters. By 2011, when adoption histories are different, both yields and input use has diverged. The one exception is basal fertilizer, which is the same between never adopters and future adopters. Thus, there is *prima facie* evidence that differences in yields on CA and TC plots are due to differences in input use and not due to differences in unobserved plot-level characteristics.

**Table 4:** Input change over time conditional on ward.

	2008			2011		
	Always adopter	Future disadopter	MW-test	Always adopter	Future disadopter	MW-test
ln (Yield) (kg/ha)	0.469 (1.190)	0.386 (1.270)		0.512 (0.939)	−0.028 (1.113)	^∗∗∗^
ln (Seed) (kg/ha)	0.089 (0.802)	0.206 (0.883)	^∗^	0.029 (0.717)	−0.054 (0.674)	^∗^
ln (Basal fertilizer) (kg/ha)	0.122 (0.799)	0.009 (0.851)		0.188 (0.912)	−0.312 (0.835)	^∗∗∗^
ln (Top fertilizer) (kg/ha)	0.201 (0.846)	0.052 (0.857)		0.363 (0.974)	−0.242 (0.940)	^∗∗∗^
Observations	162	299		86	346	

	Never adopter	Future adopter	MW test	Never adopter	Future adopter	MW-test
ln (Yield) (kg/ha)	−0.394 (1.281)	−0.239 (1.366)		−0.154 (1.188)	0.455 (1.064)	^∗∗∗^
ln (Seed) (kg/ha)	−0.172 (0.944)	−0.001 (0.831)		0.012 (0.769)	0.132 (0.857)	^∗^
ln (Basal fertilizer) (kg/ha)	−0.298 (1.198)	0.054 (0.597)		−0.127 (0.813)	0.125 (1.109)	
ln (Top fertilizer) (kg/ha)	−0.415 (1.057)	0.068 (0.778)	^∗∗^	−0.190 (0.873)	0.285 (1.157)	^∗∗∗^
Observations	446	84		656	75	

*Note*: Table displays the mean residuals, and their standard deviations in parenthesis, of output and inputs by adoption type and year. Residuals are calculated from either a regression of output on ward indicators or the input on ward indicators. In the upper panel, “Always adopters” are those plots that in every year were cultivated with CA. They are compared to “Future disadopters,” those plots under CA in 2008 but which reverted to TC in subsequent years. In the lower panel, “Never adopters” are those plots that were never cultivated using CA. They are compared to “Future adopters,” those plots under TC in 2008 but which were put under CA in subsequent years. The final column for each year presents the results of Mann-Whitney two-sample tests for differences in distribution. Results are similar if a Kolmogorov-Smirnov test is used. Significance of MW-tests are reported as ∗p < 0.1; ∗∗p < 0.05; ∗∗∗p < 0.01.

In summary, Table 3 demonstrates that a naive comparison that does not consider differences in input use would lead to the erroneous conclusion that CA, by itself, increases yields for all crops. To address this issue, we include measured inputs by crop type in our regression analysis so that we are comparing CA plots to TC plots with similar input use. A second concern is that yields under CA might respond differently from yields under TC. Fig. 3 shows that controlling for differences in access to inputs at the ward level explains most of the differences in yield response. Our use of household fixed effects controls for this and means that we are comparing yields on CA plots within a household during periods of high and low rainfall shocks to the norms a household experiences over time. A final concern is that households may choose to apply CA to certain plots based on unobservable plot-level characteristics, thus making plot-level comparisons within a household invalid. Table 4 shows that differences in yields are due to differences in input use and not differences in unobserved plot-level characteristics. As a robustness check, in subsection 5.3 we estimate yield functions using plot-level fixed effects, which allows us to compare yields under CA and TC methods for plots whose adoption status changed over the period of study.

### 4.2 Selection bias

Having established the relevant comparison group within our data, we next deal with the potential of selection bias. Because adoption of CA is not random, it is likely to be correlated with unobserved time-varying factors. In the case of Zimbabwe, CA was promoted as one element in the PRP, which also included subsidies for inputs (Mazvimavi et al., 2008). Thus, adoption rates of CA within a ward are strongly correlated with the level of input subsidies distributed in the ward (Mazvimavi and Twomlow, 2009; Ndlovu et al., 2014; Pedzisa et al., 2015b). Because the intensity of promotion and the level of subsidies changed from year-to-year, adoption of CA is neither random nor static and therefore is likely correlated with unobserved time-varying factors.

**Fig. 4:**
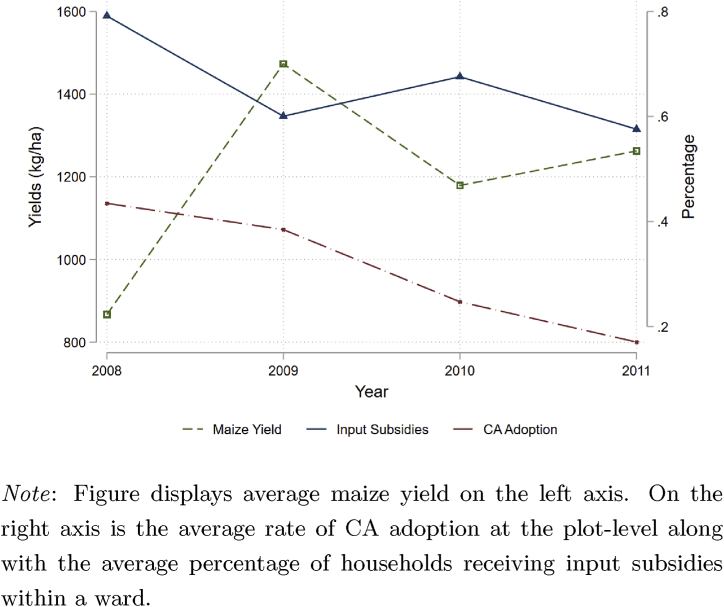
Maize yields, rates of CA adoption, and input subsidies.

To address the endogeneity of CA we instrument for plot-level adoption using the number of households in the ward that receive NGO support as part of the PRP. An appropriate instrument for this model need not be random but must be correlated with the plot-level decision to adopt CA and uncorrelated with plot yields, except through the treatment. As Fig. 4 shows, there is strong correlation between the probability that a household adopts CA and their receiving assistance from an NGO, as part of the PRP, in the form of input subsidies. According to the final report on the PRP, rural households were categorized into four standardized groups based on asset ownership, labor availability, and severity of cash constraints (Jennings et al., 2013). While the PRP provided support to 1.7 million people, those who were eligible for input subsidies, and thus most likely to adopt CA, were required to be households with land and labor but no cash. Eligibility for NGO support was well defined but due to budgetary limitations not all eligible households received support, meaning that NGOs may have targeted individual households based on unobservable characteristics.

That not all eligible households received subsidized inputs raises the concern that correlation exists among NGO targeting of households, CA adoption, and yields. To demonstrate that our instrument satisfies the exclusion restriction, we must demonstrate that any correlation between our instrument and yields occurs only through the adoption of CA or can be explicitly controlled for in our regression framework. One potential pathway of contamination is through NGO targeting of households based on time-invariant characteristics, such as those defined in the eligibility criteria of the PRP. Our use of fixed effects controls for this possibility, making the use of NGO support a plausibly valid instrument. A second potential pathway of contamination is that input subsidies likely have a direct effect on yields, making it an invalid instrument. Here we can directly control for this pathway by including the amount of seed and fertilizer applied on each plot, though we cannot control for quality of inputs. A third potential pathway of contamination is that certain wards or districts received abnormal levels of support because of local levels of land or asset ownership. While average land and asset ownership at the district-level does vary, the percentage of households in a district receiving NGO support is remarkably consistent across districts, never falling below 59 percent and never reaching above 80 percent. A final potential pathway of contamination is that NGO targeting was based on some unobservable time-varying characteristics that are directly correlated with yields. While far from definitive proof, Fig. 4 shows that there is little correlation from year-to-year between plot-level yields, CA adoption by household, and the number of households within a ward that received input subsidies as part of the PRP.^11^ The level of support that neighboring households receive is correlated with a given household receiving NGO support but does not directly impact that household's plot-level yields, especially once we control for input use, household effects, and local conditions.^12^ Thus, the number of households in the ward that receive NGO support satisfies the exclusion restriction.

### 4.3 Estimation procedure

Our primary specification follows directly from Eq. (15):

(20) $$y_{kit}=\beta_{k}h_{kit}+\theta_{k}+\phi_{k}\theta_{k}h_{kit}+x_{jkit}^{\prime }\gamma_{jk}+\tau_{i}+\delta_{t}+\varepsilon_{kit}.$$

Recall that *h*_*kit*_ is an indicator for whether crop *k* on plot *i* at time *t* is under CA cultivation and *β*_*k*_ is the associated impact of CA on yields. Note also that *θ*_*k*_ is the impact on crop *k* of rainfall shock R_*wt*_ in ward *w* at time *t*, as defined in Eq. (9). *ϕ*_*k*_ is the measure of the yield resilience of crop *k* when it is cultivated using CA and experiences rainfall shock R_*wt*_, *x*′_*jkit*_ is a vector of crop, plot, and time specific inputs, including crop-specific intercept terms, and *τ*_*i*_ is a time-invariant household effect. To our original yield function we add year dummies as additional controls.

Given that adoption of CA, *h*_*it*_, is interacted with either crop type (to give *h*_*kit*_) or with crop type and the exogenous rainfall shock (to give *θ*_*k*_*h*_*kit*_), we follow Wooldridge (2003) in instrumenting for only the potentially endogenous term. Prior to estimating Eq. (20) with two-stage least squares (2SLS), we estimate a zero-stage probit that predicts *ĥ*_*it*_ from the following latent variable model of adoption:

(21) $$h_{it}=1\left[\zeta {Z\;}_{wt}+\theta_{k}+x_{jkit}^{\prime }\alpha_{jk}+\tau_{i}+\delta_{t}+v_{it}\geq 0\right],$$

where Z_*wt*_ is number of households in ward *w* that receive NGO support at time *t*, *ζ* is the associated coefficient, and $v_{it}∼N\left(0,\sigma_{v_{it}}^{2}\right)$ and is independent of *u*_*it*_ and *τ*_*i*_. Since our zero-stage equation is nonlinear, we use a Mundlak-Chamberlain device to control for household unobservables instead of the fixed effects used in the 2SLS. This amounts to replacing *τ*_*i*_ with its linear projection onto the time averages of observable choice variables $\tau_{i}={\bar{x\;}}_{i}\lambda +c_{i}$, where $c_{i}∼N\left(0,\sigma_{c_{i}}^{2}\right)$ (Mundlak, 1978; Chamberlain, 1984). Using the Mundlak-Chamberlain device allows us to avoid the incidental variable problem created by using fixed effects in a nonlinear model while still controlling for unobservables (Wooldridge, 2010). While our zero-stage and 2SLS equations take different approaches to controlling for *τ*_*i*_, identification of *h*_*it*_ still fully relies on Z_*wt*_. Any difference that exists between the fixed effects and the Mundlak-Chamberlain device is captured in *c*_*i*_, which in expectation has zero mean. Our use of the Mundlak-Chamberlain device will be less efficient than fixed effects to the extent that $\sigma_{c_{i}}^{2}>0$, but this introduces no bias into our estimate of *h*_*it*_. Most importantly, the use of the Mundlak-Chamberlain device does not change the causal identification as it strips out time-invariant effects in the same way as using fixed effects and therefore introduces no bias into our subsequent 2SLS estimates. This has become the preferred method for controlling for time-invariant unobserved heterogeneity in non-linear equations (Ricker-Gilbert et al., 2011; Bezu et al., 2014; Verkaart et al., 2017).

We then calculate the Inverse Mills Ratio (IMR), or sample selection correction term, using *ĥ*_*it*_ and construct instruments for *h*_*kit*_ by interacting the IMR with crop type and construct instruments for *θ*_*ki*_*h*_*kit*_ by interacting the IMR with crop type and the exogenous rainfall shock. Wooldridge (2003) shows that this approach produces consistent estimates and improves on efficiency when compared to instrumenting the entire interaction term. We then use these instruments to estimate Eq. (20) using 2SLS.

This allows us to conduct several diagnostic tests for our instruments. First, we calculate the Kleibergen-Paap LM statistic, which is a test for underidentification (i.e., is the correlation between the endogenous variables and the instruments statistically different from zero). Second, we conduct two different weak instrument tests, which are a higher hurdle than the test for underidentification. The problem with weak instrument tests is that there is currently no universally accepted way in which to construct *p*-values for these statistics when 1) errors are not i.i.d. and 2) the equation is exactly identified (Bazzi and Clemens, 2013). This is because the Stock and Yogo (2005) critical values frequently used in weak instrument tests are calculated under the assumption that the errors in the relevant regression are i.i.d. and that there are at least two more overidentifying restrictions than the number of endogenous variables. We address this problem by conducting two different weak instruments tests. First, we report the Anderson and Rubin (1949) test statistic. The AR test, in the case of our exactly identified equations, is a test that the coefficients on all endogenous regressors are zero. This test is robust to weak instruments yet it lacks power in the presence of many instruments, which we have (Kleibergen and Paap, 2006). As an alternative to the AR test, we follow Bazzi and Clemens (2013) in constructing *p*-values for use in conducting weak instrument tests using the Kleibergen-Paap Wald stat, which is robust to heteroskedasticity. Because critical values have not been tabulated for the Kleibergen-Paap Wald stat, we follow the literature and apply the critical values tabulated by Stock and Yogo (2005) for the Cragg-Donald Wald stat to the Kleibergen-Paap results (Baum et al., 2007).^13^

To ensure correct hypothesis testing on our coefficients, we allow the variance structure of the error term to vary by household as well as by crop and cluster our standard errors at the household-crop level. This procedure is not without its critics. Bertrand et al. (2004) suggest that clustering at a single level is preferred to clustering at two levels. This provides two alternatives: cluster only at the crop-level or cluster only at the household-level. Given that we only have five different crops, and a large set of parameters to estimate, we are unable to directly cluster standard errors at just the crop level. Clustering at the household-level provides results qualitatively equivalent to those when we cluster at the household-crop level.^14^

## 5 Results

We present the results from a large complement of estimates in Table 5, Table 6, Table 7, Table 8. All models are estimated using the log of yield as the dependent variable and log values of measured inputs as independent variables. Hence, point estimates can be read directly as elasticities.^15^ For brevity, we only report coefficients on CA, the rainfall deviations, and the interaction terms. Estimated coefficients on measured inputs are presented in Online Appendix B.

**Table 5:** Yield function with CA as exogenous.

	(1)	(2)	(3)	(4)
*Maize*				
CA (= 1)	0.631^∗∗∗^ (0.081)	0.573^∗∗∗^ (0.081)	0.222 (0.139)	0.207 (0.137)
rainfall shock			−0.667^∗∗∗^ (0.215)	−0.904^∗∗∗^ (0.227)
CA × rainfall shock			0.872^∗∗∗^ (0.247)	0.744^∗∗∗^ (0.237)
*Sorghum*				
CA (= 1)	0.041 (0.180)	−0.051 (0.190)	−0.756^∗∗∗^ (0.271)	−0.596^∗∗^ (0.288)
rainfall shock			−1.285^∗∗∗^ (0.303)	−1.432^∗∗∗^ (0.317)
CA × rainfall shock			1.652^∗∗∗^ (0.458)	1.130^∗∗^ (0.499)
*Millet*				
CA (= 1)	−0.145 (0.359)	0.118 (0.374)	−0.994 (0.709)	−0.711 (0.736)
rainfall shock			−1.094^∗∗∗^ (0.306)	−1.521^∗∗∗^ (0.319)
CA × rainfall shock			1.492 (0.940)	1.475 (1.171)
*Groundnut*				
CA (= 1)	0.299^∗∗^ (0.142)	0.323^∗∗^ (0.144)	−0.326 (0.287)	0.130 (0.279)
rainfall shock			−0.403^∗^ (0.208)	−0.489^∗∗^ (0.228)
CA × rainfall shock			1.106^∗∗^ (0.493)	0.331 (0.456)
*Cowpea*				
CA (= 1)	−0.035 (0.241)	0.194 (0.250)	−0.844^∗^ (0.466)	−0.362 (0.460)
rainfall shock			−1.053^∗∗^ (0.452)	−1.216^∗∗∗^ (0.448)
CA × rainfall shock			1.688^∗^ (0.905)	1.114 (0.878)
Household FE	No	Yes	No	Yes
Observations	7643	7643	7643	7643
*R*^2^	0.899	0.922	0.900	0.923

*Note*: Dependent variable is log of yield. Though not reported, all specifications include crop-specific inputs and intercept terms, and year dummies. See Table B1 in the Online Appendix for coefficient estimates of crop-specific inputs. Column (1) excludes the rainfall variable as well as household fixed effects. Column (2) excludes the rainfall variable but includes household fixed effects. Column (3) includes the rainfall variable and its interaction with CA but excludes household fixed effects. Column (4) includes both the rainfall variable, its interaction with CA, and household fixed effects. Standard errors clustered by household and crop are reported in parentheses (∗p < 0.1; ∗∗p < 0.05; ∗∗∗p < 0.01).

**Table 6:** Zero-stage probit.

	(1)	(2)	(3)	(4)
Number of HH in ward with NGO support	0.010^∗∗∗^ (0.001)	0.009^∗∗∗^ (0.001)	0.010^∗∗∗^ (0.001)	0.009^∗∗∗^ (0.001)
Household MCD	No	Yes	No	Yes
Observations	7643	7643	7643	7643
Log Likelihood	−3153	−3110	−3143	−3100

*Note*: Dependent variable is an indicator for whether or not CA was used on the plot. Though not reported, all probit regressions include crop-specific inputs and intercept terms, and year dummies. Column (1) excludes the rainfall variable as well as the Mundlak-Chamberlain device (MCD). Column (2) excludes the rainfall variable but includes the MCD. Column (3) includes the rainfall variable but excludes the MCD. Column (4) includes both the rainfall variable and the MCD. Standard errors clustered by household and crop are reported in parentheses (∗p < 0.1; ∗∗p < 0.05; ∗∗∗p < 0.01).

**Table 7:** Yield function with CA as endogenous.

	(1)	(2)	(3)	(4)
*Maize*				
CA (= 1)	13.404 (10.916)	−1.939^∗^ (1.122)	15.697 (18.025)	−2.853^∗∗^ (1.141)
rainfall shock			0.569 (2.198)	−1.675^∗∗∗^ (0.353)
CA × rainfall shock			1.335 (2.896)	2.628^∗∗∗^ (0.704)
*Sorghum*				
CA (= 1)	19.230 (13.886)	−0.275 (1.498)	24.937 (24.052)	−0.171 (1.603)
rainfall shock			−0.940 (0.823)	−1.476^∗∗∗^ (0.353)
CA × rainfall shock			−2.555 (4.717)	0.899 (0.904)
*Millet*				
CA (= 1)	32.388 (30.713)	−4.551 (3.402)	49.502 (64.115)	−3.784 (3.808)
rainfall shock			0.654 (2.163)	−1.715^∗∗∗^ (0.341)
CA × rainfall shock			−12.938 (30.199)	3.357 (2.279)
*Groundnut*				
CA (= 1)	14.997 (10.282)	0.050 (1.199)	16.999 (16.357)	−0.272 (1.324)
rainfall shock			−1.659^∗^ (0.886)	−0.950^∗∗∗^ (0.265)
CA × rainfall shock			2.423 (2.895)	1.433∗ (0.832)
*Cowpea*				
CA (= 1)	10.530 (8.312)	−0.938 (1.147)	12.451 (14.121)	−1.564 (1.349)
rainfall shock			−0.061 (1.643)	−1.606^∗∗∗^ (0.485)
CA × rainfall shock			0.775 (3.789)	2.123 (1.404)
Household FE	No	Yes	No	Yes
Observations	7643	7643	7643	7643
Log Likelihood	−24,462	−16,378	−25,914	−16,192
Kleibergen-Paap LM stat	1.773	26.89^∗∗∗^	1.028	33.86^∗∗∗^
Anderson-Rubin Wald stat	37.38^∗∗∗^	19.47^∗∗∗^	75.09^∗∗∗^	35.88^∗∗∗^
Kleibergen-Paap Wald stat	0.355	4.593^∗∗∗^	0.102	2.955^∗∗∗^

*Note*: Dependent variable is log of yield. Though not reported, all specifications include crop-specific inputs and intercept terms, and year dummies. See Table B2 in the Online Appendix for coefficient estimates of crop-specific inputs. In each regression the adoption of CA is treated as endogenous and is instrumented with the Inverse Mills Ratio (IMR) calculated from the predicted values of the zero-stage probits reported in Table 6. The CA × rainfall shock term is also treated as endogenous and instrumented using the interaction of the IMR and the rainfall shock term. The null hypothesis of the Kleibergen-Paap LM test is that the rank condition fails (i.e., the first-stage equation is underidentified). The null hypothesis of the Anderson-Rubin Wald test is that the coefficients on the endogenous regressors in the structural equation are jointly equal to zero (i.e., the instruments in the first-stage equation are weak). The null hypothesis of the Kleibergen-Paap Wald test is that a *t*-test at the 5*%* significance level on the coefficients of the endogenous regressors rejects no more than 25*%* of the time (i.e., the instruments in the first-stage equation are weak). Standard errors clustered by household and crop are reported in parentheses (∗p < 0.1; ∗∗p < 0.05; ∗∗∗p < 0.01).

**Table 8:** Yield function with rain shortage or surplus.

	(1)	(2)	(3)	(4)
*Maize*				
CA (= 1)	−0.466 (1.379)	−2.657^∗^ (1.378)	−1.893 (1.472)	3.393 (3.156)
rainfall shortage	0.089 (0.400)		−1.076^∗∗^ (0.481)	−1.411^∗∗^ (0.695)
CA × rainfall shortage	0.147 (0.744)		2.098^∗∗^ (0.987)	3.522^∗∗^ (1.597)
rainfall surplus		−1.619^∗∗∗^ (0.304)	−1.884^∗∗∗^ (0.358)	−0.444 (0.637)
CA × rainfall surplus		2.012^∗∗∗^ (0.513)	2.931^∗∗∗^ (0.657)	1.953^∗∗^ (0.845)
*Sorghum*				
CA (= 1)	−0.262 (1.464)	−1.211 (2.042)	−0.512 (1.780)	5.808 (4.791)
rainfall shortage	−1.079^∗∗∗^ (0.389)		−1.702^∗∗∗^ (0.454)	−2.235^∗∗∗^ (0.769)
CA × rainfall shortage	2.437^∗^ (1.263)		2.356^∗^ (1.326)	3.654^∗^ (1.977)
rainfall surplus		−0.550 (0.342)	−1.214^∗∗∗^ (0.377)	−0.531 (0.533)
CA × rainfall surplus		−0.369 (1.008)	0.464 (0.987)	−0.144 (1.507)
*Millet*				
CA (= 1)	−2.913 (2.890)	−3.914 (3.404)	−2.671 (3.293)	12.779 (12.606)
rainfall shortage	−0.508 (0.610)		−1.604^∗∗^ (0.658)	−1.438 (0.927)
CA × rainfall shortage	−0.136 (1.576)		1.930 (2.344)	−4.318 (8.524)
rainfall surplus		−1.287^∗∗∗^ (0.356)	−1.689^∗∗∗^ (0.320)	−0.427 (0.584)
CA × rainfall surplus		2.726^∗^ (1.548)	3.170 (2.202)	−0.775 (7.605)
*Groundnut*				
CA (= 1)	0.359 (1.159)	−1.519 (1.550)	−0.266 (1.499)	4.764 (3.664)
rainfall shortage	−1.125^∗∗∗^ (0.318)		−1.229^∗∗∗^ (0.338)	−2.298^∗∗^ (0.934)
CA × rainfall shortage	0.240 (0.772)		0.405 (0.984)	1.499 (1.570)
rainfall surplus		0.036 (0.297)	−0.170 (0.281)	0.506 (0.465)
CA × rainfall surplus		0.056 (0.899)	−0.205 (0.985)	−0.607 (1.422)
*Cowpea*				
CA (= 1)	−0.742 (1.141)	−0.929 (1.415)	−1.188 (1.439)	1.829 (3.818)
rainfall shortage	−0.628 (0.477)		−1.493^∗∗∗^ (0.553)	−2.468^∗∗^ (1.221)
CA × rainfall shortage	3.171^∗∗∗^ (1.008)		3.763^∗∗∗^ (1.272)	6.006^∗∗^ (2.558)
rainfall surplus		−1.103^∗∗^ (0.482)	−1.492^∗∗∗^ (0.557)	−0.970 (1.002)
CA × rainfall surplus		−0.286 (1.309)	1.234 (1.624)	3.148 (2.543)
Type of Effect	Household FE	Household FE	Household FE	Plot MCD
Observations	7643	7643	7643	5004
Log Likelihood	−15,908	−16,406	−15,902	−12,268
Kleibergen-Paap LM stat	22.96^∗∗∗^	23.02^∗∗∗^	25.23^∗∗∗^	3.790^∗∗^
Anderson-Rubin Wald stat	22.10^∗∗^	29.68^∗∗∗^	44.79^∗∗∗^	38.53^∗∗∗^
Kleibergen-Paap Wald stat	1.930^∗^	1.989^∗^	1.444	0.251

*Note*: Dependent variable is log of yield. Though not reported, all specifications include crop-specific inputs and intercept terms, and year dummies. See Table B4 in the Online Appendix for coefficient estimates of crop-specific inputs. In each regression the adoption of CA is treated as endogenous and is instrumented with the Inverse Mills Ratio (IMR) calculated from the predicted values of zero-stage probits which are presented in the Online Appendix, Table B3. The CA × rainfall shortage and CA × rainfall surplus terms are also treated as endogenous and instrumented using the interaction of the IMR and the rainfall terms. The null hypothesis of the Kleibergen-Paap LM test is that the rank condition fails (i.e., the first-stage equation is underidentified). The null hypothesis of the Anderson-Rubin Wald test is that the coefficients on the endogenous regressors in the structural equation are jointly equal to zero (i.e., the instruments in the first-stage equation are weak). The null hypothesis of the Kleibergen-Paap Wald test is that a *t*-test at the 5*%* significance level on the coefficients of the endogenous regressors rejects no more than 25*%* of the time (i.e., the instruments in the first-stage equation are weak). Standard errors clustered by household and crop are reported in parentheses (∗p < 0.1; ∗∗p < 0.05; ∗∗∗p < 0.01).

### 5.1 Main results

We first focus on the results presented in Table 5 in which CA is treated as exogenous. While it is unlikely that the decision to adopt CA is uncorrelated with the time-varying error term, these results are informative as they allow us to directly compare our estimates with previous literature on the correlation between CA and yields. Column (1) presents a simple yield function that lacks both our rainfall variable and household fixed effects. Results in column (2) come from the same regression but with fixed effects to control for time-invariant household unobservables. The correlation between CA and yields is positive and significant for both maize and groundnut with and without household fixed effects. In all other crop cases CA has no statistically significant association with yields.

Adding deviations from average rainfall and its interaction with CA tells a very different story. Columns (3) and (4) present point estimates of the more flexible yield function with and without household fixed effects. Focusing on the fixed effects results in column (4), CA by itself no longer increases yields for any crop and appears to be correlated with lower yields for sorghum. Exposure to a rainfall shock decreases yields for all crops. When we examine the interaction terms, we find that CA is correlated with higher yields for maize and sorghum during periods of rainfall stress. For millet, groundnut, and cowpea CA has no statistically significant association with yields, regardless of rainfall levels.

The results in columns (1) and (2) of a positive correlation between maize yields and CA are similar to the results presented in much of the previous literature (Kassie et al., 2009; Mazvimavi and Twomlow, 2009; Kassie et al., 2010; Teklewold et al., 2013a, b; Brouder and Gomez-Macpherson, 2014; Ndlovu et al., 2014; Abdulai, 2016; Manda et al., 2016). These positive and statistically significant correlations are often interpreted as demonstrating that CA increases yields compared to TC and are used to justify the continued promotion of CA (Giller et al., 2009). However, similar to Arslan et al. (2015), we find that this result is not robust to the inclusion of rainfall measures. Additionally, we expect these results to be biased due to correlation between the decision to adopt and the error term.

Table 6 presents results from the zero-stage probit regressions. For all four specifications the number of households in the ward that receive NGO support is positive and significantly correlated with the choice to adopt CA. In Table 7 we present results similar to those in Table 5 but controlling for the endogeneity of CA. Our instruments pass both the underidentification and weak instrument tests, but only in regressions with household fixed effects. Because of this, our preferred specification is column (4) because it includes CA-rainfall interaction terms and simultaneously controls for unobserved shocks through the IV and unobserved heterogeneity through household fixed effects. We find that CA, by itself, decreases yields on maize and provides no significant advantage over TC for the other crops. Similar to the results in column (4) of Table 5, we find that rainfall deviations reduce yields on all crops.

While the lack of impact of CA on yields is discouraging, it is not the full story.^16^ When we examine the interaction between CA and rainfall we find that CA increases yields in times of rainfall stress for maize and groundnut. For all crops, except sorghum, the coefficients on the CA terms are of a larger magnitude than in the regressions that treat CA as exogenous. Once we control for the endogeneity of the adoption decision, the coefficients on CA tend to be more negative while the coefficients on the interaction terms tend to be more positive. The bias generated from not controlling for the endogeneity of CA appears to underestimate the impact, either positive or negative, of CA on yields. Having controlled for the bias, we conclude that smallholder farmers in Zimbabwe who cultivate their crops using CA practices receive higher yields compared to conventional farmers but only in times of rainfall stress. Online Appendix C presents the outcomes from a number of robustness checks on our main results.

### 5.2 Alternative rainfall measures

While our main results provide evidence that CA cultivation during deviations from average rainfall helps mitigate crop loss, our measure of rainfall deviation is agnostic to whether or not the shock is from surplus rainfall or a shortage of rainfall. While proponents claim that CA will have a positive impact on yields in both abnormally wet and abnormally dry conditions, there is reason to believe that CA may be more effective in one situation compared to the other, depending on the crop in question.

The first three columns in Table 8 present results from regressions which treat CA as endogenous and include household fixed effects. Column (1) shows results from the regression with the rainfall shock measured as a shortage as in Eq. (18). Column (2) replaces the rainfall shortage with a rainfall surplus as in Eq. (19). Column (3) uses both rainfall shortage and surplus measures. Instruments in all three regressions pass the underidentification test and the AR weak instrument test. Only instruments in the regressions from column (1) and (2) pass the Kleibergen-Paap weak instrument test. Despite this, the specification in column (3) is our preferred one because it includes the full range of data and allows the impact of CA to vary based on the type of rainfall event and what crop is being cultivated.

Focusing on column (3), rainfall shortages have a negative and significant impact on yields for all crops, while rainfall surpluses have a negative and significant impact on yields for all crops, except groundnut. In average rainfall periods, the use of CA does not have a significant impact on any crop. Examining the interaction terms, the use of CA improves maize yields both when rainfall is above average and during times of drought. For sorghum and cowpea, CA improves yields during times of drought but not surplus rainfall. CA has no specific impact in mitigating losses from either surpluses or shortfalls of rain for millet and groundnut.

In summary, we find that during periods of average rainfall, CA typically has no impact on yields compared to TC. Furthermore, the coefficient on CA is generally negative, suggesting that if CA has any impact on yields it is to reduce them compared to TC. This is in marked contrast to much of the previous literature, which finds a positive correlation between CA and yields. We believe this difference is due to previous studies failing to control for the multiple sources of endogeneity in the CA adoption decision. Second, during seasons that experience above or below average rainfall, CA mitigates yield losses due to these deviations. Maize and groundnut yields are more resilient under CA. Third, when we allow for rainfall shortages to impact yields differently from surplus rainfall we find that only maize yields are consistently more resilient under CA than under TC. Online Appendix D tests the robustness of these conclusions using a number of different rainfall specifications.^17^ In general, CA either has a positive impact on yields in times of stress or it has no impact at all. We conclude that while CA may not improve yields during average seasons, and may even decrease yields, production using CA is more resilient, especially for maize, when rainfall shocks occur.

### 5.3 Plot level controls

One potential concern with our previous results is that the choice to adopt CA may not be driven by unobserved household characteristics but instead by unobserved plot-level characteristics. Given that CA is promoted as a technology to halt and reverse land degradation, households may apply CA on plots where they know soil quality is poor. If this is the case, we would expect yields on CA plots to be systematically lower than yields on other plots. Alternatively, though the reasoning is less clear, households might apply CA on plots where they know soil quality is good.

Our panel covers only four seasons with households initially adopting CA in one to four years prior to data collection. Since it takes anywhere between ten and fifteen seasons for CA to significantly increase soil organic matter (Giller et al., 2009), we assume that plot characteristics, such as soil quality, are time-invariant in our data. If this is not the case, our estimates will be biased downward. We employ panel data methods to control for the correlation between the decision to adopt and unobserved plot characteristics. However, there might also be time-variant shocks that influence the decision to put a specific plot under CA instead of influencing the household-level decision to adopt. To control for potential time-invariant shocks we again use instrumental variables.

Columns (4) in Table 8 presents results from a regression designed to control for both sources of plot-level endogeneity. We restrict the sample to plots that we observe more than once over the study period. This reduces our sample size to 5, 004 but allows us to directly control for unobserved heterogeneity at the plot-level. However, due to issues of collinearity we are unable to use plot-level fixed effects and instead implement the Mundlak-Chamberlain device in the zero-stage and 2SLS regressions. We find that our results for all crops, when controlling for endogeneity at the household-level (column (3)), are robust in our plot-level endogenous CA regression. CA by itself has no impact on yields and CA continues to build resilience for maize, sorghum, and cowpea. Similar to our previous results, we find that CA has no specific impact in mitigating losses from either surplus or shortfalls of rain for millet and groundnut.

### 5.4 Returns to CA

To provide some intuition on the size of impact CA has on yields we calculate predicted returns to adoption at various levels of rainfall as in Eq. (16). Using the results in column (3) of Table 8 (which controls for endogeneity of adoption and household fixed effects), we multiply the coefficient on the CA-rainfall interaction terms by the realized values of the rainfall shortages or surpluses. We then sum these values along with the coefficient on the CA-only term. We calculate predicted returns for each crop as well as for an average across crops.

**Fig. 5:**
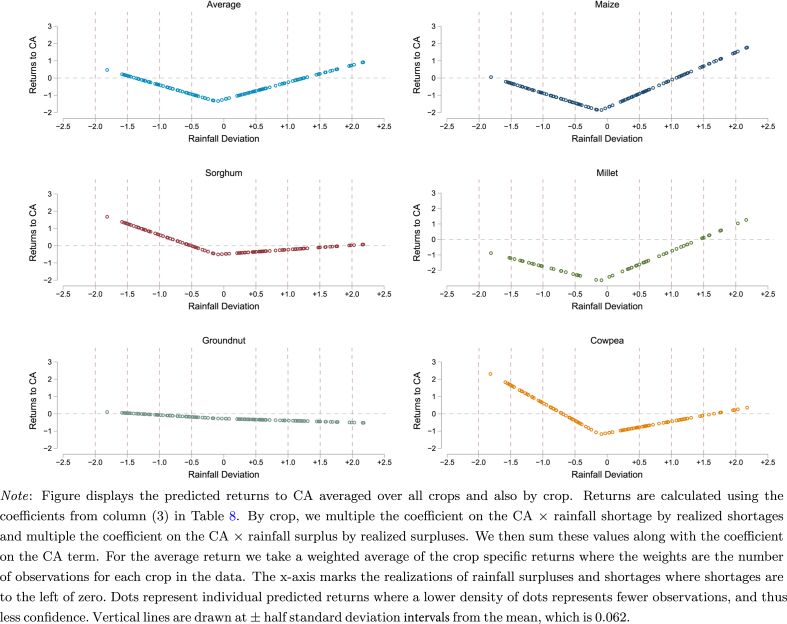
Predicted returns to CA by crop.

Fig. 5 graphs the returns to CA across the realized values of rainfall surpluses and shortages. For maize, the returns to CA are positive only when rainfall is one standard deviation above the average. In seasons where cumulative rainfall is below one standard deviation, the returns to CA practices are negative. The returns to using CA to cultivate sorghum are positive for almost any shortage in rainfall while they are close to zero for above average rainfall. For millet, the returns to CA are rarely ever positive, though unlike sorghum, returns are positive when rainfall is well above average. The returns to CA cultivation of groundnut are near zero, regardless of rainfall. Finally, for cowpea, returns to CA are similar to sorghum, though the negative effect of CA is more pronounced.

Taking a weighted average across crops, where the weights are the number of observations for each crop in the data, we can describe the returns to CA for a typical smallholder household in the multi-cropping environment of Zimbabwe. Households would need to experience rainfall shortages greater than one and a half standard deviations away from the mean or rainfall surpluses greater than one standard deviation away from the mean before the returns to CA would become positive. During seasons where rainfall was within this range, the average returns to CA would be negative and households would be better off using TC practices. Examining seasonal rainfall data from 1997 to 2015 across the 45 wards in our sample, 65 percent of seasons have fallen within this ‘normal’ range, making the returns to CA negative. Only 35 percent of the time was rainfall either low enough or high enough that the returns to CA would be positive.

To provide a sense of the economic returns to CA adoption in Zimbabwe, we also calculate predicted revenue to both CA and TC practices. First, we predict *ŷ*_CA_ and *ŷ*_TC_ for CA and TC plots from the regression presented in column (3) of Table 8. Second, we create a counterfactual value for each plot by subtracting the estimated contribution of CA ($\hat{\beta }$ and $\hat{\phi }\theta$) from *ŷ*_CA_ and adding the estimated contribution of CA to *ŷ*_TC_. This allows us to compare yields on a plot with the predict yields had the plot being cultivated using an alternative method. Third, we assign a monetary value to yields for each crop using published data from the Government of Zimbabwe's Agricultural Marketing Authority.^18^ Finally, we graph the revenue associated with each cultivation practice on each plot, holding input use constant, at different realizations of rainfall surpluses and shortages using kernel-weighted local polynomial smoothing, with confidence intervals drawn at 90 percent.

**Fig. 6:**
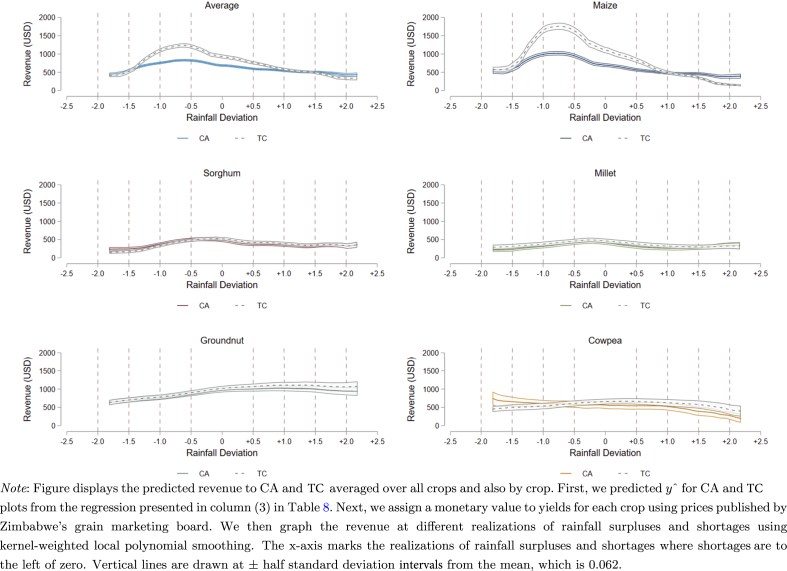
Predicted Revenue to CA and non-CA by Crop.

The results of this “back of the envelope” approach to calculating revenue are encouraging for smallholder agricultural production in Zimbabwe. At market prices, farming generates significant revenue for all crops and both cultivation methods. Whether this is sufficient revenue to allow households to make a profit is difficult to determine because of the difficulty in valuing non-market purchased inputs, such as land, household labor, or recycled seeds. With these caveats in mind, the curves in Fig. 6 tell a fairly consistent story, particularly when interpreted in combination with Fig. 5. On average, and for maize and cowpea, CA cultivation generates less revenue than TC over a large portion of the rainfall distribution. For sorghum, millet, and groundnut, CA also tends to generate less revenue, though these differences are frequently not statistically significant. Where CA produces more revenue than TC is at the far ends of the distribution, when rainfall is more than one and a half standard deviations away from the mean. Research on other ‘climate-smart’ agricultural technologies has demonstrated the importance of immediate and consistent positive returns in order to sustain adoption (Chabé-Ferret and Subervie, 2013; Damon et al., 2015). The low levels of adoption, and high disadoption rates, of CA in Zimbabwe are likely a result of the technology providing low returns over much of the rainfall distribution. We conclude that CA may not be an appropriate technology for all of Zimbabwe, or any region of Sub-Saharan Africa where rainfall is not highly variable. Rather, policy should target CA for households living in areas prone to frequent severe drought or flooding.

## 6 Conclusions and policy implications

Conservation agriculture has been widely promoted as a way for smallholder farmers in Sub-Saharan Africa to increase yields while also making yields more resilient to changing climate conditions. Using four years of panel data from Zimbabwe, we find evidence that contradicts the first claim but supports the later claim. We estimate a large compliment of yield functions that include rainfall shocks, household fixed effects, and control for the endogeneity of the choice to adopt CA practices. In all these cases we find that, where CA has a significant impact on yields, it is always to reduce yields compared to TC practices during periods of average rainfall. When we consider yields during rainfall shocks, we find that yields tend to be more resilient under CA cultivation then under TC practices. Overall, returns to CA are only positive for those households that experience high variability in rainfall patterns. We conclude that previous econometric analysis that found a positive correlation between CA and yields was most likely due to a failure to control for unobserved heterogeneity among households and selection bias in the choice to adopt CA. This conclusion comes with the caveat that our data cover only four years. In the long run, CA may indeed have a positive impact on yield. To our knowledge, no long-run observational data set exists on which this hypothesis can be tested.

Two policy recommendations can be drawn from our analysis. First, our results help address the empirical puzzle of low CA adoption rates in Sub-Saharan Africa. We find that, over the four year study period, CA had either a negative impact or no impact at all on yields during periods of average rainfall. Smallholder farmers tend to be risk averse, and CA is often associated with increased labor demand and the need for purchased fertilizer inputs. Given that returns to CA can be negative, especially for maize, it makes sense that smallholders have been hesitant to undertake the added risk and cost of CA practices. We find no evidence at the plot-level that CA is associated with yield increases and therefore conclude that households’ decision to not adopt, or disadopt as in the case of Zimbabwe, is most likely rational in the short-term. Policy to promote CA among smallholders should acknowledge this point and take steps to manage expectations regarding the short-term and long-term benefits of CA.

Second, CA can be effective in mitigating yield loss in environments with increased weather risk. Climate change threatens to disrupt normal rainfall patterns by reducing the duration and frequency of rainfall (prolonged droughts) and also by increasing the intensity of rainfall. We find that in both cases (abnormally high and abnormally low rainfall) yields are often more resilient under CA then under TC. This insight provides a way forward for the promotion of CA practices among smallholders. Policy should be designed to focus on CA's potential benefits in mitigating risk due to changing rainfall patterns.

We conclude that CA is indeed an example of ‘climate smart’ agriculture to the extent that a changing climate will result in more abnormal rainfall patterns and CA appears effective in mitigating yield loss due to deviations in rainfall. Such a conclusion does not imply that CA is a sustainable approach to agriculture for all farmers, or even for most farmers, living in Sub-Saharan Africa. This is because in periods of normal rainfall the returns to CA are negative, at least in the short run. In order to test the long-term benefits of CA on yields through improved soil fertility, future research should focus on establishing long-run observational datasets on CA practices. The challenge here is to convince enough farmers to consistently adopt costly agricultural practices that may in the short-term cost them, absent the realization of extreme rainfall events.

## Footnotes

1 This last benefit comes, in the case of sub-optimal rainfall, through the use of planting basins to achieve minimum tillage, in combination with mulching, which increases water infiltration and moisture conservation (Thierfelder and Wall, 2009). In the case of excess rainfall, the retention of crop residue reduces water-driven soil erosion (Schuller et al., 2007).

2 Recent agronomic research has shed doubt on the validity of the claim that CA sequesters significant amounts of carbon in soil (Baker et al., 2007; Piccoli et al., 2016; Powlson et al., 2016).

3 Our use of the term “adoption” refers to the use (as opposed to the *first time* use) of CA on a plot. Similarly, “disadoption” refers to the use of conventional cultivation techniques on a plot that was previously cultivated using CA.

4 Wards are the smallest administrative district in rural Zimbabwe.

5 See Online Appendix A for a more detailed discussion of year-over-year changes in the household data.

6 The rainy season in Zimbabwe runs from November to March, with households planting immediately ahead of the first rains and harvesting in May or June.

7 For more detail regarding seasonal variation in rainfall see Online Appendix A. For results from robustness checks using various subsets of years see Online Appendix D.

8 For each crop-cultivation pair we first test for normality of the data using the Shapiro-Wilk test. In every case we reject the null that the data is normally distributed. Because of this, we rely on the Mann-Whitney (MW) test instead of the standard *t*-test to determine if differences exist within crops across cultivation practices. Unlike the *t*-test, the MW test does not require the assumption of a normal distribution. In the context of summary statistics we also prefer the MW test to the Kolmogorov-Smirnov (KS) test, since the MW test is a test of location while the KS test is a test for shape. Results using the KS test are equivalent to those obtained from the MW test.

9 In addition to the possibility that quantity of input use varies by location, there is also the possibility that quality of input use varies. While the data lacks direct measures of quality, it does contain information about the source of inputs. There does not appear to be large differences in the source of fertilizer based on whether or not the plot was cultivated using CA. We do find that newly purchased seed tends to be used on TC plots, but any bias introduced by differences in seed quality would make our estimates of yield resilience due to CA an underestimate.

10 Similar to our approach in Fig. 3, we first regress each variable on ward indicators in order to control for differences in access to inputs across wards. The values displayed in each cell of the table are residuals from these regressions.

11 The correlation coefficient between CA and plot yields is ρ = 0.1585 while the correlation coefficient between our IV and plot yields is ρ = 0.0018.

12 One may be concerned with potential spillover effects, in that a household that adopts CA due to NGO support might result in increased yields for a neighbor. For the case of CA in Zimbabwe this is unlikely for two reasons. First, the practices that constitute CA are location specific, in that use of planting basins or residue or rotation on one plot will have no impact on the productivity of another plot. Second, even if this was not the case, households in the survey are relatively dispersed throughout wards, meaning that the plots of one household are not contiguous with neighboring households.

13 For recent uses of weak instrument tests in applications where the Stock and Yogo (2005) critical values do not directly apply, see Sanderson and Windmeijer (2016) and Bun and Harrison (2018).

14 To address the issue of too few crops for clustering, we estimated crop-specific yield functions with household fixed effects as a system of equations. This approach allows for correlation among crop-specific error terms, though it implicitly imposes the assumption that observations are independent if they are in the same household (Greene, 2011). We also estimated our preferred specification but clustered errors at just the household level.

15 Given the prevalence of zero values in the input data, and to a lesser extent in the output data, we use the inverse hyperbolic sine transformation to convert levels into logarithmic values.

16 It is prudent to remember that in many cases the positive yield impacts of CA only appear after ten to fifteen years of CA cultivation (Giller et al., 2009, 2011). Given that our panel only covers four years, it may be that households have yet to reap the benefits of CA practices.

17 We also conducted tests using satellite temperature data in place of and in combination with rainfall data. Online Appendix A briefly discusses these results.

18 A strong caveat to this process is that we use price data from May 2018 in order to value production from the survey, which occurred between 2008 and 2011. The primary reason for this is that the surveys took place during and immediately after the period of hyperinflation in Zimbabwe and therefore did not collect price data but focused on the physical measurement of inputs and output. Because of these data limitations we are unable to calculate returns to adoption in terms of profit using prices from the survey period.

## References

[bib1] Abdulai A.N. (2016). Impact of conservation agriculture technology on household welfare in Zambia. Agric. Econ..

[bib2] Anderson T., Rubin H. (1949). Estimation of the parameters of single equation in a complete system of stochastic equations. Ann. Math. Stat..

[bib3] Andersson J.A., D'Souza S. (2014). From adoption claims to understanding farmers and contexts: a literature review of conservation agriculture (CA) adoption among smallholder farmers in Southern Africa. Agric. Ecosyst. Environ..

[bib4] Arslan A., McCarthy N., Lipper L., Asfaw S., Catteneo A. (2014). Adoption and intensity of adoption of conservation farming practices in Zambia. Agric. Ecosyst. Environ..

[bib5] Arslan A., McCarthy N., Lipper L., Asfaw S., Catteneo A., Kokwe M. (2015). Climate smart agriculture? Assessing the adaptation implications in Zambia. J. Agric. Econ..

[bib6] Baker J.M., Ochsner T.E., Venterea R.T., Griffis T.J. (2007). Tillage and soil carbon sequestration — what do we really know?. Agric. Ecosyst. Environ..

[bib7] Barrett C.B., Moser C.M., McHugh O.V., Barison J. (2004). Better technology, better plots, or better farmers? Identifying changes in productivity and risk among Malagasy rice farmers. Am. J. Agric. Econ..

[bib8] Baum C.F., Schaffer M.E., Stillman S. (2007). Enhanced routines for instrumental variables/GMM estimation and testing. STATA J..

[bib9] Bazzi S., Clemens M.A. (2013). Blunt instruments: avoiding common pitfalls in identifying the causes of economic growth. Am. Econ. J. Macroecon..

[bib10] Bertrand M., Duflo E., Mullainathan S. (2004). How much should we trust differences-in-differences estimates?. Q. J. Econ..

[bib11] Bezu S., Kassie G.T., Shiferaw B., Ricker-Gilbert J. (2014). Impact of improved maize adoption on welfare of farm households in Malawi: a panel data analysis. World Dev..

[bib12] Brouder S.M., Gomez-Macpherson H. (2014). The impact of conservation agriculture on smallholder agricultural yields: a scoping review of the evidence. Agric. Ecosyst. Environ..

[bib13] Bun M.J., Harrison T.D. (2018). OLS and IV estimation of regression models including endogenous interaction terms. Econom. Rev..

[bib14] Chabé-Ferret S., Subervie J. (2013). How much green for the buck? Estimating additional and windfall effects of French agro-environmental schemes by DID-matching. J. Environ. Econ. Manag..

[bib15] Chamberlain G., Griliches Z., Intriligator M.D. (1984). Panel data.

[bib16] Damon M., Graff Zivin J., Thirumurthy H. (2015). Health shocks and natural resource management: evidence from Western Kenya. J. Environ. Econ. Manag..

[bib17] Di Falco S., Chavas J.-P. (2008). Rainfall shocks, resilience, and the effects of crop biodiversity on agroecosystem productivity. Land Econ..

[bib18] FAO (2013). Climate-smart Agriculture Sourcebook.

[bib19] FAO, IFAD, UNICEF, WFP, WHO (2017). The State of Food Security and Nutrition in the World 2017: Building Resilience for Peace and Food Security.

[bib20] Funk C., Peterson P., Landseld M., Pedreros D., Verdin J., Shukla S., Husak G., Roland J., Harrison L., Hoell A., Michaelsen J. (2015). The climate hazards infrared precipitation with stations – a new environmental record for monitoring extremes. Sci. Data.

[bib21] Giller K.E., Corbeels M., Nyamangara J., Triomphe B., Affholder F., Scopel E., Tittonell P. (2011). A research agenda to explore the role of conservation agriculture in African smallholder farming systems. Field Crop. Res..

[bib22] Giller K.E., Witter E., Corbeels M., Tittonell P. (2009). Conservation agriculture and smallholder farming in Africa: the heretics' view. Field Crop. Res..

[bib23] Greene W.H. (2011). Econometric Analysis.

[bib24] Jennings M., Kayondo A., Kagoro J., Nicholson K., Blight N., Gayfer J. (2013). Impact Evaluation of the Protracted Relief Programme II, Zimbabwe.

[bib25] Just R.E., Pope R.D. (1978). Stochastic specification of production functions and economic implications. J. Econom..

[bib26] Kassie M., Teklewold H., Jaletab M., Marenyab P., Erenstein O. (2015). Understanding the adoption of a portfolio of sustainable intensification practices in eastern and southern Africa. Land Use Pol..

[bib27] Kassie M., Teklewold H., Marenya P., Jaleta M., Erenstein O. (2015). Production risks and food security under alternative technology choices in Malawi: application of a multinomial endogenous switching regression. J. Agric. Econ..

[bib28] Kassie M., Zikhali P., Manjur K., Edwards S. (2009). Adoption of sustainable agricultural practices: evidence from a semi-arid region of Ethiopia. Nat. Resour. Forum.

[bib29] Kassie M., Zikhali P., Pender J., Köhlin G. (2010). The economics of sustainable land management practices in the Ethiopian highlands. J. Agric. Econ..

[bib30] Kleibergen F., Paap R. (2006). Generalized reduced rank tests using the singular value decomposition. J. Econom..

[bib31] Lal R., Stewart B. (2010). Food Security and Soil Quality.

[bib32] Lipper L., Thornton P., Campbell B.M., Baedeker T., Braimoh A., Bwalya M., Caron P., Cattaneo A., Garrity D., Henry K., Hottle R., Jackson L., Jarvis A., Kossam F., Mann W., McCarthy N., Meybeck A., Neufeldt H., Remington T., Sen P.T., Sessa R., Shula R., Tibu A., Torquebiau E.F. (2014). Climate-smart agriculture for food security. Nat. Clim. Change.

[bib33] Lobell D.B., Schlenker W., Costa-Roberts J. (2011). Climate trends and global crop production since 1980. Science.

[bib34] Manda J., Alene A.D., Gardebroek C., Kassie M., Tembo G. (2016). Adoption and impacts of sustainable agricultural practices on maize yields and incomes: evidence from rural Zambia. J. Agric. Econ..

[bib35] Mazvimavi K., Twomlow S. (2009). Socioeconomic and institutional factors influencing adoption of conservation farming by vulnerable households in Zimbabwe. Agric. Syst..

[bib36] Mazvimavi K., Twomlow S., Belder P., Hove L. (2008). An Assessment of the Sustainable Adoption of Conservation Farming in Zimbabwe. Global Theme on Agroecosystems.

[bib37] Michler J.D., Shively G.E. (2015). Land tenure, tenure security and farm efficiency: panel evidence from the Philippines. J. Agric. Econ..

[bib38] Morton J.F. (2007). The impact of climate change on smallholder and subsistence agriculture. Proc. Natl. Acad. Sci. U. S. A..

[bib39] Mundlak Y. (1978). On the pooling of time series and cross section data. Econometrica.

[bib40] Ndlovu P.V., Mazvimavi K., An H., Murendo C. (2014). Productivity and efficiency analysis of maize under conservation agriculture in Zimbabwe. Agric. Syst..

[bib41] Pannell D.J., Llewellyn R.S., Corbeels M. (2014). The farm-level economics of conservation agriculture for resource-poor farmers. Agric. Ecosyst. Environ..

[bib42] Pedzisa T., Rugube L., Winter-Nelson A., Baylis K., Mazvimavi K. (2015). Abandonment of conservation agriculture by smallholder farmers in Zimbabwe. J. Sustain. Dev..

[bib43] Pedzisa T., Rugube L., Winter-Nelson A., Baylis K., Mazvimavi K. (2015). The intensity of adoption of conservation agriculture by smallholder farmers in Zimbabwe. Agrekon.

[bib44] Piccoli I., Chiarini F., Carletti P., Furlan L., Lazzaro B., Nardi S., Berti A., Sartori L., Dalconi M., Morari F. (2016). Disentangling the effects of conservation agriculture practices on the vertical distribution of soil organic carbon. evidence of poor carbon sequestration in North-Eastern Italy. Agric. Ecosyst. Environ..

[bib45] Pittelkow C.M., Liang X., Linquist B.A., van Groenigen K.J., Lee J., Lundy M.E., van Gestel N., Six J., Ventera R.T., van Kessel C. (2015). Productivity limits and potentials of the principles of conservation agriculture. Nature.

[bib46] Powlson D.S., Stirling C.M., Thierfelder C., White R.P., Jat M. (2016). Does conservation agriculture deliver climate change mitigation through soil carbon sequestration in tropical agro-ecosystems?. Agric. Ecosyst. Environ..

[bib47] Ricker-Gilbert J., Jayne T.S., Chirwa E. (2011). Subsidies and crowding out: a double-hurdle model of fertilizer demand in Malawi. Am. J. Agric. Econ..

[bib48] Sanderson E., Windmeijer F. (2016). A weak instrument *F*-test in linear IV models with multiple endogenous variables. J. Econom..

[bib49] Schlenker W., Lobell D.B. (2010). Robust negative impacts of climate change on African agriculture. Environ. Resour. Lett..

[bib50] Schuller P., Walling D.E., Sepúlveda A., Castillo A., Pino I. (2007). Changes in soil erosion associated with the shift from conventional tillage to a no-tillage system, documented using ^137^Cs measurements. Soil Tillage Res..

[bib51] Smith P., Powlson D.S., Glendining M.J., Smith J.U. (1998). Preliminary estimates of the potential for carbon mitigation in european soils through no-till farming. Global Change Biol..

[bib52] Stock J.H., Yogo M., Andrews D.W., Stock J.H. (2005). Testing for weak instruments in linear IV regression. Identification and Inference for Econometric Models: Essays in Honor of Thomas Rothenberg.

[bib53] Suri T. (2011). Selection and comparative advantage in technology adoption. Econometrica.

[bib54] Teklewold H., Kassie M., Shiferaw B. (2013). Adoption of multiple sustainable agricultural practices in rural Ethiopia. J. Agric. Econ..

[bib55] Teklewold H., Kassie M., Shiferaw B., Köhlin G. (2013). Cropping system diversification, conservation tillage, and modern seed adoption in Ethiopia: impacts on household income, agrochemical use, and demand for labor. Ecol. Econ..

[bib56] Thierfelder C., Wall P.C. (2009). Effects of conservation agriculture techniques on infiltration and soil water content in Zambia and Zimbabwe. Soil Tillage Res..

[bib57] Verkaart S., Munyua B.G., Mausch K., Michler J.D. (2017). Welfare impacts of improved chickpea adoption: a pathway for rural development in Ethiopia?. Food Pol..

[bib58] Ward P.S., Shively G.E. (2015). Migration and land rental as responses to income shocks in rural China. Pac. Econ. Rev..

[bib59] Wheeler T., von Braun J. (2013). Climate change impacts on global food security. Science.

[bib60] Wooldridge J.M. (2003). Further results on instrumental variables estimation of average treament effects in the correlated random coefficient model. Econ. Lett..

[bib61] Wooldridge J.M. (2010). Econometric Analysis of Cross Section and Panel Data.

