# Supplementary material for: Conservation agriculture and climate resilience
Source: J Environ Econ Manage. 2019 Jan;93:148–69. doi: 10.1016/j.jeem.2018.11.008 (PMC6358000; doi:10.1016/j.jeem.2018.11.008)
Supplement: Multimedia component 1 [file mmc1.pdf]

Online-Only Appendix to "Conservation Agriculture and Climate Resilience"

A Crop Production & Rainfall Histories in Zimbabwe

This appendix contains information regarding the distribution of observations in our unbalanced panel, historical rainfall patterns in Zimbabwe, and a discussion of potential concerns about the correlation between weather variables and output.

Our analysis relies on unbalanced panel data. While in most observational data sets, a lack of balance in a panel is a result of attrition, in our case the lack of balance is a result of adding households to the survey over time. Because of this, we have observations from more households (and more plots) in later years. Since our sample is random, we do not expect there to be any differences in the types of households added to the survey in later years. However, weather events are not evenly distributed across time. Thus, to some extent, our overall results are driven by the weather that was occurring in 2010 and 2011 because that is when a majority of the data comes from. Figures A1-A3 provide the reader with an idea to what extent our results are driven by the unbalanced distribution across years.

Figure A1 presents a bar graph of the frequency of plot-level observations for each crop in each year. The number of observations of plots with maize growing on them increases consistently from year-to-year. A similar pattern exists for observations of plots with groundnut and cowpea. While the entire sample size increases each year, the number of plots with sorghum and millet does not always increase. Figure A2 graphs the percentage of observations for each crop in each year. Here we see that while the total number of observations changed from year-to-year, the percentage breakdown for each crop was fairly stable. Maize makes up between 47 and 54 percent of plot-level observations each year. Sorghum makes up between 12 and 19 percent of plot-level observations each year. Similarly narrow bands of variation exist for the other three crops. Taken together, Figures A1 and A2 demonstrate that while the number of observations increases over time, the make-up of our sample, at least with regards to crops, does not change much over time.

Despite the fair amount of consistency in regards to the make-up of crops in our sample over time, we do see variation in crop yields. In Figure A3 we draw kernel densities for all crops by year and each individual crop by year. Here we see that 2009 tended to be the best year in terms of yields. Yields tended to be lower (and more variable) in 2011. Obviously, a number of factors effect yields, and our econometric analysis is designed to identify the impact of CA adoption, rainfall, and their joint impacts, while controlling for inputs and household characteristics. What Figure A3 makes clear, though, is that yields in 2011 were lower than in previous years and since there are more observations from 2011 what occurred in that year will play a large roll in determining our results.

Beyond understanding when our data comes from, it is important to know what sort of weather occurred in each year and if that weather was in line with historical weather trends. An initial concern with using rainfall data to predict crop yield is that one has chosen the correct window within which to measure rainfall. As discussed in the paper, rainfall in Zimbabwe is often of high intensity but low duration and frequency, meaning that monthly averages may be more appropriate than our seasonal averages used in the paper. To test for this, we examine the impact of both seasonal and monthly deviations in rainfall on crop output (see Table A1). We find little empirical evidence that late onset of rainfall or mid-season dry spells reduce yields. Rainfall in the first month

of the growing season only impacts maize and groundnut yields while deviations in the mid-season months tend to only affect maize, groundnut, and cowpea. By contrast, for all five crops, deviations in seasonal rainfall have a significant and negative impact on crop yields. Based on these results we conclude that our use of seasonal deviations is a strong proxy for rainfall induced stresses to agricultural production.

Having determined that our seasonal measure of rainfall is a strong predictor of crop yield, a second concern is that the data contain sufficient variation in realized rainfall over the four year study period. To determine this we draw the distribution of cumulative seasonal rainfall (see Figure A4). The distribution forms a fairly broad band, with half the observations (49 percent) falling outside half a standard deviation of the mean and nearly a third of observations (29 percent) falling outside one standard deviations of the mean.

Incidentally, we also explored using satellite temperature data from Sheffield et al. (2014) in our analysis. However, unlike the rainfall data there was insufficient variation in the temperature data. We calculated deviations in average seasonal temperature from long-run averages along with growing degree days (GDDs). For both measures there was almost no variation over space or over time. In the areas of Zimbabwe where the data come from, temperature throughout the growing season did not fluctuate greatly during our time frame, making it difficult to identify the impact of temperature on crops without a longer panel.

A final concern is that because we have only four years of data, one outlier year might play an out-sized role in our results. To determine how rainfall differed across the four years, Table A2 presents summary statistics of the rainfall shocks by year. 2008 and 2010 were wet years relative to the 15 year average while 2011 was a dry year. 2009 was variable, with some regions experiencing surplus rainfall while others experienced rainfall shortage. From Figure 2 in the paper, it is clear that our data come from a period of above average rainfall, though the final year of 2011 was drier than average. 2011 is the sixth driest season in the past 15 years while 2008 was the fifth rainiest season in the past 15 years. Based on the evidence in Table A2 and Figure 2 we conclude that the rainfall draws from the four years of household data are typical of historic rainfall patterns in Zimbabwe, though on the slightly rainier side.

B Full Results from Yield Function

This appendix contains full specifications and zero-stage probit results for the models presented in an abbreviated form in the paper. In all specifications we include controls for crop-specific inputs. These inputs include logs of basal fertilizer application, of top application of fertilizer, of seed, and of land area. Since our primary focus is the impact of CA and rainfall shocks on yields, we treat these variables as exogenous controls and refrained from reporting the relevant point estimates in the paper. For those interested in the full specifications, the following tables will be of interest.

C Additional Robustness Checks

In this appendix we verify the robustness of our main results to changes in our underlying specification.

A first concern is that our results rely on the functional form of our zero-stage regression. To check this, we replace our zero-stage probit using the Mundlak-Chamberlain device with a zero-stage Tobit using fixed effects. Results are consistent regardless of how we specify the zero-stage

selection decision (see Tables C1 and C2).

A second concern is that our indicator for CA is capturing something else and thereby creating spurious correlation between CA and yields. We can check to see if this is the case by constructing placebo tests or pseudo-treatments.[19] To do this, we divide the sample using a similar logic behind our sample split in Table 4 from the paper. We begin by constructing two different comparison groups. The first defines a pseduo-treatment in which "treated" plots are those that will, sometime in the future (2009, 10, or 11), have CA but at the moment are cultivated using conventional methods. As an example, if a plot was cultivated using traditional methods in 2008 and 2009 but was a CA plot in 2010 and 2011, we mark the plot as "treated" and keep only the observations from 2008 and 2009. The "control" plots are those that never have CA and are always cultivated using conventional methods.

The second comparison group defines a pseduo-treatment in which "treated" plots are those that were, sometime in the past (2008, 09, or 10), CA plots but at the moment are cultivated using conventional methods. As an example, if a plot was a CA plot in 2008 but was cultivated using traditional methods in 2009-11, we mark the plot as "treated" and keep only the observations from 2009-11. These are compared to "control" plots which never have CA and are always cultivated using conventional methods.

By constructing our pseudo-treatments in this way, we are comparing plots that are always conventionally cultivated with plots that are currently being conventionally cultivated but where in the past, or will be in the future, CA plots. We expect that CA will have no significant effect on yields, nor will the CA-rainfall interaction have a significant effect. We do, however, expect the rainfall shock to reduce yields.

Results from these regressions are presented in Table C3. Column (1) presents results from our first comparison group, where "treatment" is being a future CA plot and "control" is always being a conventional plot. CA and the CA-rainfall interaction has no impact on yields for maize, millet, groundnut, and cowpea. Only in the case of sorghum does our placebo test generate significant results. Column (2) presents results from our second comparison group, where "treatment" is being a CA plot in the past and "control" is always being a conventional plot. Here CA and CA-rainfall are never significant. Across the two regressions, the rainfall shock term reduces yields in 7 of 10 cases.

Based on the regression results in Tables C2 and C3 we conclude that our main results are robust to changes in the underlying specification and that our estimates of the impact of CA are casually identified.

D Alternative Rainfall Measures

In this appendix we test the robustness of our main conclusions in the paper to changes in our rainfall data.

First, one might be concerned that, because we have only four years of data (i.e., four rainfall draws), one outlier year might be driving the results.[20] To see if this is the case, we estimate the yield function for all years, dropping one year at a time. Table D1 reports results when we treat CA as endogenous and use our single rainfall shock variable. For purposes of comparison, column (1) reproduces results from Table 7 in the paper. Column (2) removes data from 2011, column

[19]We thank an anonymous reviewer for suggesting this approach.

[20]We thank an anonymous reviewer for bringing this concern to our attention.

(3) removes data from 2010, column (4) removes data from 2009, and column (5) removes data from 2008. Because we have an unbalanced panel, the number of observations vary between $5,300$ and $7,643$. Despite this, the signs and significance levels on coefficients in columns (3) - (5) are very similar to those in column (1), which uses all four years. Additionally, the coefficients from sorghum, millet, groundnut, and cowpea are very similar across all columns. Where we do see differences is for maize in column (2), where we drop the relatively dry year of 2011.

Using our preferred specification from the paper (which includes both shortages and surplus) we estimate the yield function using all three year combinations. The results are presented in Table D2. Again, the only place where we see differences in estimates are for maize in column (2), particularly for CA, which now has a positive impact on maize yields, and CA interacted with rainfall shortage, which now reduces maize yields.

It is clear that our results for maize are driven by rainfall shortages in the 2011 year. However, we are okay with this fact because 2011 is the only year where we see significant rainfall shortage in maize growing regions. Over 60 percent of our observations for maize during rainfall shortage come from 2011. Estimating the yield function without data from 2011 amounts to asking our data to do out of sample predictions for maize during dry spells using a sample skewed towards wet years.

A second concern might be that our results rely on our chosen measure of rainfall shock. To test this, we develop two new, non-linear measures of rainfall shocks. In the first we replace any deviation in rainfall that is within $\pm$ one standard deviation of the mean (0.476) with a zero. Thus, any realized value that is $0.202 \leq \mathrm{R}_{wt} \leq 0.749$ is set to zero. In the second we replace any deviation in rainfall that is within $\pm$ one half of a standard deviation of the mean (0.476) with a zero. Thus, any realized value that is $0.339 \leq \mathrm{R}_{wt} \leq 0.612$ is set to zero. These changes to our rainfall shock term do not have a material effect on our results (see Table D3)

Figure A1: Plot Observations by Crop and Year

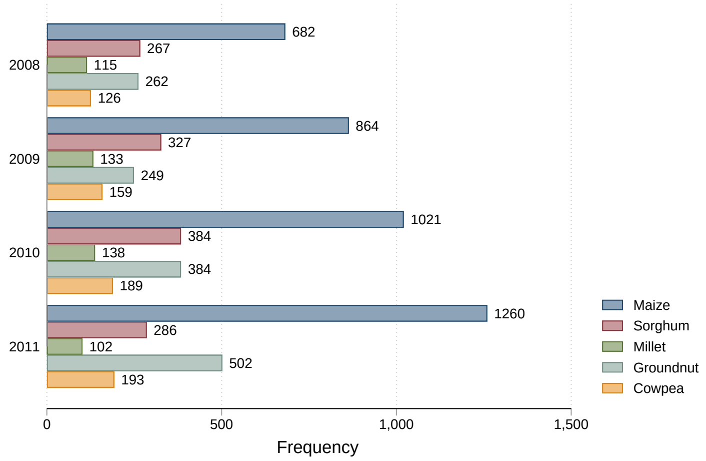

Note: Figure displays the frequency of plot-level observations for each crop in each year.

Figure A2: Percentage of Crops in Each Year

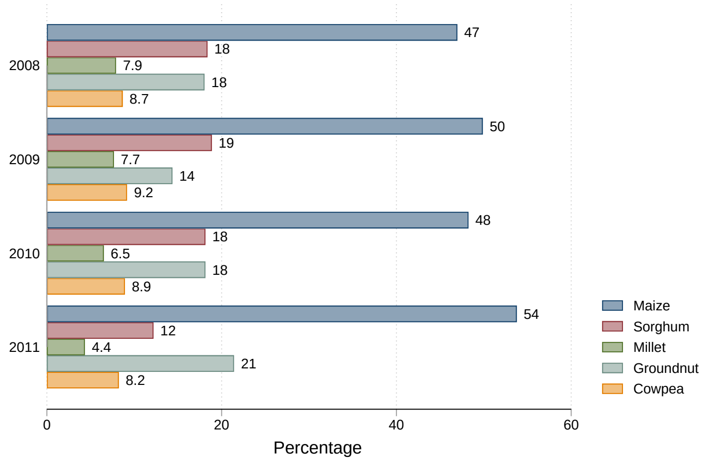

Note: Figure displays the percentage of plot-level observations growing each crop in each year.

Figure A3: Yield Distributions by Crop and Year

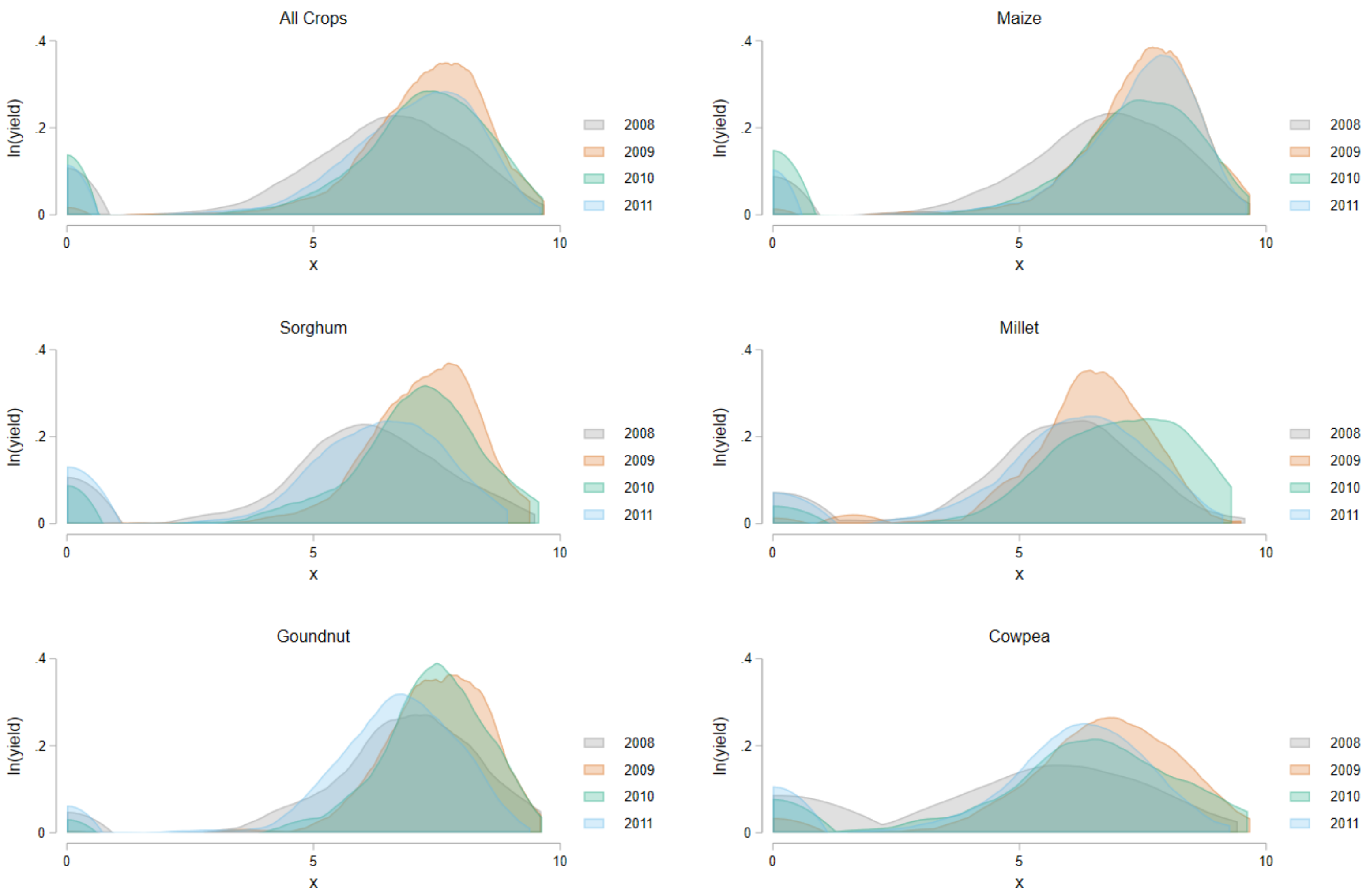

Note: Figure displays kernel densities of yields for each crop in each year.

Figure A4: Distribution of Seasonal Rainfall by Ward

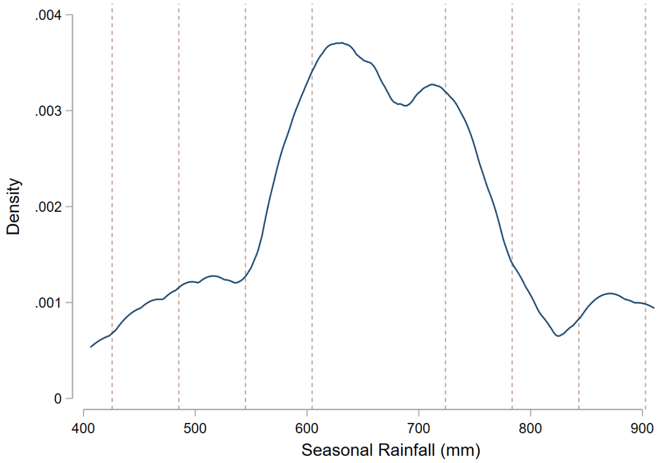

Note: Figure displays the kernel density of cumulative seasonal rainfall from each ward for the four year study period (2008-2011). Vertical lines are drawn at ± half, one, one half, and two standard deviations from the mean, which is 664mm. Data comes from the CHIRPS database (Funk et al., 2015).

Table A1: Yield Function with Rainfall Deviations

	Maize (1)	Sorghum (2)	Millet (3)	Groundnut (4)	Cowpea (5)
Panel A: Deviation in Seasonal Rainfall					
Seasonal deviation	−0.631***	−1.125***	−1.389***	−0.407*	−0.913**
	(0.192)	(0.269)	(0.312)	(0.212)	(0.399)
Panel B: Deviation in Monthly Rainfall					
Oct deviation	0.324*	−0.262	0.023	0.534***	0.129
	(0.196)	(0.212)	(0.302)	(0.199)	(0.314)
Nov deviation	−0.213*	−0.056	−0.076	0.154	−0.028
	(0.111)	(0.176)	(0.194)	(0.127)	(0.238)
Dec deviation	−0.199*	−0.122	−0.535*	0.226*	0.338
	(0.114)	(0.222)	(0.309)	(0.136)	(0.265)
Jan deviation	−0.571***	0.119	−0.267	0.251	0.418*
	(0.167)	(0.176)	(0.261)	(0.153)	(0.235)
Feb deviation	−1.054***	−0.320	−0.254	−0.683***	−2.011***
	(0.213)	(0.274)	(0.358)	(0.259)	(0.443)
Mar deviation	−1.001***	−0.701**	−0.587	−0.764***	0.112
	(0.187)	(0.322)	(0.506)	(0.242)	(0.386)
Apr deviation	−0.295***	0.104	−0.372***	−0.154	−0.015
	(0.092)	(0.108)	(0.140)	(0.105)	(0.155)

Note: Coefficients are presented in columns based on crop type in order to minimize space. Panel A reports results from a single regression with log yield as the dependent variable and deviations in cumulative seasonal rainfall as the variable of interest (Observations = $7,643$; $R^2 = 0.923$). Panel B reports results from a single regression with log yield as the dependent variable and deviations in cumulative monthly rainfall as the variables of interest (Observations = $7,643$; $R^2 = 0.926$). Though not reported, both specifications include crop-specific CA adoption terms, crop-specific inputs, crop-specific intercept terms, year dummies, and household fixed effects. Standard errors clustered by household and crop are reported in parentheses (*p<0.1; **p<0.05; ***p<0.01).

Table A2: Rainfall Shocks by Year

	2008	2009	2010	2011
Rainfall shock	0.682	0.487	0.351	0.453
	(0.289)	(0.267)	(0.225)	(0.231)
Rainfall shortage	0.000	0.282	0.028	0.442
	(0.000)	(0.257)	(0.065)	(0.246)
Rainfall surplus	0.682	0.205	0.323	0.011
	(0.289)	(0.347)	(0.253)	(0.042)
number of observations	1,452	1,732	2,116	2,343

Note: Columns in the table display means of the data by year with standard deviations in parenthesis.

Table B1: Yield Function with CA as Exogenous

	(1)	(2)	(3)	(4)
Maize				
CA (= 1)	0.631***	0.573***	0.222	0.207
	(0.081)	(0.081)	(0.139)	(0.137)
rainfall shock			−0.667***	−0.904***
			(0.215)	(0.227)
CA × rainfall shock			0.872***	0.744***
			(0.247)	(0.237)
ln(basal)	0.118***	0.079***	0.120***	0.085***
	(0.025)	(0.024)	(0.025)	(0.023)
ln(top)	0.321***	0.270***	0.312***	0.261***
	(0.028)	(0.026)	(0.028)	(0.025)
ln(seed)	0.250***	0.248***	0.236***	0.233***
	(0.075)	(0.074)	(0.075)	(0.073)
ln(area)	−0.449***	−0.496***	−0.442***	−0.483***
	(0.061)	(0.064)	(0.061)	(0.063)
Sorghum				
CA (= 1)	0.041	−0.051	−0.756***	−0.596**
	(0.180)	(0.190)	(0.271)	(0.288)
rainfall shock			−1.285***	−1.432***
			(0.303)	(0.317)
CA × rainfall shock			1.652***	1.130**
			(0.458)	(0.499)
ln(basal)	0.038	0.015	0.037	0.020
	(0.089)	(0.070)	(0.091)	(0.070)
ln(top)	0.107	0.130**	0.106	0.124**
	(0.066)	(0.060)	(0.065)	(0.059)
ln(seed)	0.395***	0.428***	0.396***	0.426***
	(0.112)	(0.119)	(0.111)	(0.118)
ln(area)	−0.527***	−0.560***	−0.500***	−0.530***
	(0.085)	(0.098)	(0.085)	(0.098)
Millet				
CA (= 1)	−0.145	0.118	−0.994	−0.711
	(0.359)	(0.374)	(0.709)	(0.736)
rainfall shock			−1.094***	−1.521***
			(0.306)	(0.319)
CA × rainfall shock			1.492	1.475
			(0.940)	(1.171)
ln(basal)	0.230**	0.180	0.191*	0.136
	(0.105)	(0.127)	(0.102)	(0.127)
ln(top)	−0.233*	−0.126	−0.216*	−0.109
	(0.123)	(0.130)	(0.122)	(0.125)
ln(seed)	0.221	0.264*	0.258*	0.298**
	(0.156)	(0.148)	(0.153)	(0.144)
ln(area)	−0.451***	−0.584***	−0.465***	−0.601***

Continued on Next Page. . .

Table B1 – Continued

	(1)	(2)	(3)	(4)
	(0.135)	(0.129)	(0.134)	(0.127)
Groundnut				
CA (= 1)	0.299**	0.323**	−0.326	0.130
	(0.142)	(0.144)	(0.287)	(0.279)
rainfall shock			−0.403*	−0.489**
			(0.208)	(0.228)
CA × rainfall shock			1.106**	0.331
			(0.493)	(0.456)
ln(basal)	0.075	−0.007	0.081	−0.008
	(0.076)	(0.067)	(0.075)	(0.067)
ln(top)	−0.085	−0.039	−0.052	−0.030
	(0.076)	(0.065)	(0.075)	(0.066)
ln(seed)	0.560***	0.470***	0.558***	0.470***
	(0.076)	(0.076)	(0.076)	(0.076)
ln(area)	−0.490***	−0.614***	−0.487***	−0.608***
	(0.077)	(0.074)	(0.077)	(0.074)
Cowpea				
CA (= 1)	−0.035	0.194	−0.844*	−0.362
	(0.241)	(0.250)	(0.466)	(0.460)
rainfall shock			−1.053**	−1.216***
			(0.452)	(0.448)
CA × rainfall shock			1.688*	1.114
			(0.905)	(0.878)
ln(basal)	0.306***	0.106	0.298***	0.108
	(0.112)	(0.095)	(0.112)	(0.095)
ln(top)	−0.134	−0.085	−0.127	−0.082
	(0.112)	(0.099)	(0.115)	(0.102)
ln(seed)	0.440***	0.385**	0.459***	0.395**
	(0.164)	(0.163)	(0.165)	(0.163)
ln(area)	−0.541***	−0.652***	−0.550***	−0.660***
	(0.108)	(0.114)	(0.109)	(0.115)
Household FE	No	Yes	No	Yes
Observations	7,643	7,643	7,643	7,643
R^2	0.899	0.922	0.900	0.923

Note: Dependent variable is log of yield. All specifications include crop-specific intercept terms and year dummies. Column (1) excludes rainfall variables as well as household fixed effects. Column (2) excludes rainfall variables but includes household fixed effects. Column (3) includes the rainfall shock and its interaction with CA but excludes household fixed effects. Column (4) includes both the rainfall shock, its interaction with CA, and household fixed effects. Standard errors clustered by household and crop are reported in parentheses (*p<0.1; **p<0.05; ***p<0.01).

Table B2: Yield Function with CA as Endogenous

	(1)	(2)	(3)	(4)
Maize				
CA (= 1)	13.404	−1.939*	15.697	−2.853**
	(10.916)	(1.122)	(18.025)	(1.141)
rainfall shock			0.569	−1.675***
			(2.198)	(0.353)
CA × rainfall shock			1.335	2.628***
			(2.896)	(0.704)
ln(basal)	−0.632	0.219***	−0.789	0.219***
	(0.639)	(0.069)	(0.993)	(0.063)
ln(top)	−0.283	0.404***	−0.433	0.360***
	(0.527)	(0.065)	(0.795)	(0.057)
ln(seed)	1.819	−0.052	2.189	−0.017
	(1.347)	(0.164)	(2.105)	(0.150)
ln(area)	−0.006	−0.608***	0.101	−0.568***
	(0.429)	(0.090)	(0.630)	(0.082)
Sorghum				
CA (= 1)	19.230	−0.275	24.937	−0.171
	(13.886)	(1.498)	(24.052)	(1.603)
rainfall shock			−0.940	−1.476***
			(0.823)	(0.353)
CA × rainfall shock			−2.555	0.899
			(4.717)	(0.904)
ln(basal)	−0.840	0.043	−0.978	0.023
	(0.678)	(0.106)	(1.006)	(0.096)
ln(top)	−3.028	0.150	−3.788	0.060
	(2.260)	(0.236)	(3.640)	(0.220)
ln(seed)	0.246	0.395***	0.236	0.400***
	(0.317)	(0.113)	(0.387)	(0.113)
ln(area)	1.480	−0.551***	1.936	−0.467***
	(1.453)	(0.180)	(2.290)	(0.166)
Millet				
CA (= 1)	32.388	−4.551	49.502	−3.784
	(30.713)	(3.402)	(64.115)	(3.808)
rainfall shock			0.654	−1.715***
			(2.163)	(0.341)
CA × rainfall shock			−12.938	3.357
			(30.199)	(2.279)
ln(basal)	−1.721	0.453*	−2.112	0.228
	(2.080)	(0.237)	(2.943)	(0.196)
ln(top)	−4.778	0.547	−6.297	0.209
	(4.456)	(0.530)	(7.398)	(0.482)
ln(seed)	−1.215	0.424*	−1.737	0.371*
	(1.547)	(0.225)	(2.612)	(0.198)
ln(area)	3.501	−1.163***	4.771	−0.858**
	(3.877)	(0.437)	(6.379)	(0.402)
Groundnut				
CA (= 1)	14.997	0.050	16.999	−0.272
	(10.282)	(1.199)	(16.357)	(1.324)
rainfall shock			−1.659*	−0.950***
			(0.886)	(0.265)
CA × rainfall shock			2.423	1.433*
			(2.895)	(0.832)
ln(basal)	−1.137	0.001	−1.370	−0.036
	(0.893)	(0.114)	(1.369)	(0.106)
ln(top)	−1.966	−0.008	−2.362	−0.031
	(1.336)	(0.159)	(2.133)	(0.158)
ln(seed)	1.090***	0.566***	1.197**	0.561***
	(0.398)	(0.084)	(0.591)	(0.086)
ln(area)	0.405	−0.654***	0.603	−0.610***
	(0.676)	(0.103)	(1.040)	(0.096)
Cowpea				
CA (= 1)	10.530	−0.938	12.451	−1.564
	(8.312)	(1.147)	(14.121)	(1.349)
rainfall shock			−0.061	−1.606***
			(1.643)	(0.485)
CA × rainfall shock			0.775	2.123
			(3.789)	(1.404)
ln(basal)	0.049	0.146	−0.014	0.134
	(0.296)	(0.099)	(0.399)	(0.096)
ln(top)	−1.999	0.100	−2.385	0.047

Continued on Next Page...

Table B2 – Continued

	(1)	(2)	(3)	(4)
	(1.477)	(0.205)	(2.297)	(0.197)
ln(seed)	−0.231	0.433***	−0.362	0.446***
	(0.592)	(0.167)	(0.901)	(0.168)
ln(area)	0.517	−0.776***	0.745	−0.741***
	(0.852)	(0.163)	(1.319)	(0.159)
Household FE	No	Yes	No	Yes
Observations	7,643	7,643	7,643	7,643
Log Likelihood	-24,462	-16,378	-25,914	-16,192
Kleibergen-Paap LM stat	1.773	26.89***	1.028	33.86***
Anderson-Rubin Wald stat	37.38***	19.47***	75.09***	35.88***
Kleibergen-Paap Wald stat	0.355	4.593***	0.102	2.955***

Note: Dependent variable is log of yield. All specifications include crop-specific intercept terms and year dummies. In each regression the adoption of CA is treated as endogenous and is instrumented with the Inverse Mills Ratio (IMR) calculated from the predicted values of the zero-stage probits reported in Table 6. The CA × rainfall shock term is also treated as endogenous and instrumented using the interaction of the IMR and the rainfall shock term. The null hypothesis of the Kleibergen-Paap LM test is that the rank condition fails (i.e., the first-stage equation is underidentified). The null hypothesis of the Anderson-Rubin Wald test is that the coefficients on the endogenous regressors in the structural equation are jointly equal to zero (i.e., the instruments in the first-stage equation are weak). The null hypothesis of the Kleibergen-Paap Wald test is that a *t*-test at the 5% significance level on the coefficients of the endogenous regressors rejects no more than 25% of the time (i.e., the instruments in the first-stage equation are weak). Standard errors clustered by household and crop are reported in parentheses (*p<0.1; **p<0.05; ***p<0.01).

Table B3: Zero-Stage Probit with Rain Shortage or Surplus

	(1)	(2)	(3)	(4)
Number of HH in ward	0.009***	0.009***	0.009***	0.010***
with NGO support	(0.001)	(0.001)	(0.001)	(0.002)
Type of Effect	Household MCD	Household MCD	Household MCD	Plot MCD
Observations	7,643	7,643	7,643	5,004
Log Likelihood	-3,107	-3,102	-3,096	-2,102

Note: Dependent variable is an indicator for whether or not CA was used on the plot. Though not reported, all probit regressions include crop-specific inputs and intercept terms, and year dummies. Column (1) excludes the rainfall variable as well as the Mundlak-Chamberlain device (MCD). Column (2) excludes the rainfall variable but includes the MCD. Column (3) includes the rainfall variable but excludes the MCD. Column (4) includes both the rainfall variable and the MCD. Standard errors clustered by household and crop are reported in parentheses (*p<0.1; **p<0.05; ***p<0.01).

Table B4: Yield Function with Rain Shortage or Surplus

	(1)	(2)	(3)	(4)
Maize				
CA (= 1)	−0.466	−2.657*	−1.893	3.393
	(1.379)	(1.378)	(1.472)	(3.156)
rainfall shortage	0.089		−1.076**	−1.411**
	(0.400)		(0.481)	(0.695)
CA × rainfall shortage	0.147		2.098**	3.522**
	(0.744)		(0.987)	(1.597)
rainfall surplus		−1.619***	−1.884***	−0.444
		(0.304)	(0.358)	(0.637)
CA × rainfall surplus		2.012***	2.931***	1.953**
		(0.513)	(0.657)	(0.845)
ln(basal)	0.131	0.248***	0.166**	−0.115
	(0.080)	(0.084)	(0.081)	(0.172)
ln(top)	0.319***	0.397***	0.305***	0.138
	(0.074)	(0.078)	(0.073)	(0.108)
ln(seed)	0.122	−0.053	0.100	0.699**
	(0.187)	(0.193)	(0.185)	(0.342)
ln(area)	−0.532***	−0.595***	−0.518***	−0.462***
	(0.091)	(0.096)	(0.089)	(0.150)
Sorghum				
CA (= 1)	−0.262	−1.211	−0.512	5.808
	(1.464)	(2.042)	(1.780)	(4.791)
rainfall shortage	−1.079***		−1.702***	−2.235***
	(0.389)		(0.454)	(0.769)
CA × rainfall shortage	2.437*		2.356*	3.654*
	(1.263)		(1.326)	(1.977)
rainfall surplus		−0.550	−1.214***	−0.531
		(0.342)	(0.377)	(0.533)
CA × rainfall surplus		−0.369	0.464	−0.144
		(1.008)	(0.987)	(1.507)
ln(basal)	0.018	0.114	0.037	−0.190
	(0.107)	(0.108)	(0.101)	(0.264)
ln(top)	0.079	0.311	0.093	−0.671
	(0.249)	(0.289)	(0.257)	(0.553)
ln(seed)	0.396***	0.407***	0.405***	0.508***
	(0.113)	(0.112)	(0.112)	(0.194)
ln(area)	−0.518***	−0.663***	−0.510***	−0.008
	(0.182)	(0.195)	(0.179)	(0.420)
Millet				
CA (= 1)	−2.913	−3.914	−2.671	12.779
	(2.890)	(3.404)	(3.293)	(12.606)
rainfall shortage	−0.508		−1.604**	−1.438
	(0.610)		(0.658)	(0.927)
CA × rainfall shortage	−0.136		1.930	−4.318
	(1.576)		(2.344)	(8.524)
rainfall surplus		−1.287***	−1.689***	−0.427
		(0.356)	(0.320)	(0.584)
CA × rainfall surplus		2.726*	3.170	−0.775
		(1.548)	(2.202)	(7.605)
ln(basal)	0.342*	0.275	0.161	−0.524
	(0.207)	(0.198)	(0.177)	(0.671)
ln(top)	0.312	0.364	0.098	−1.148
	(0.455)	(0.483)	(0.424)	(1.090)
ln(seed)	0.358*	0.380*	0.325*	0.022
	(0.208)	(0.198)	(0.186)	(0.360)
ln(area)	−0.957**	−0.935**	−0.738**	0.809
	(0.383)	(0.391)	(0.353)	(1.229)
Groundnut				
CA (= 1)	0.359	−1.519	−0.266	4.764
	(1.159)	(1.550)	(1.499)	(3.664)
rainfall shortage	−1.125***		−1.229***	−2.298**
	(0.318)		(0.338)	(0.934)
CA × rainfall shortage	0.240		0.405	1.499
	(0.772)		(0.984)	(1.570)
rainfall surplus		0.036	−0.170	0.506
		(0.297)	(0.281)	(0.465)
CA × rainfall surplus		0.056	−0.205	−0.607
		(0.899)	(0.985)	(1.422)
ln(basal)	−0.012	0.119	0.027	−0.256
	(0.110)	(0.128)	(0.110)	(0.291)
ln(top)	−0.072	0.191	0.014	−0.551
	(0.158)	(0.182)	(0.172)	(0.435)

Continued on Next Page...

Table B4 – Continued

	(1)	(2)	(3)	(4)
ln(seed)	0.563***	0.516***	0.543***	0.891***
	(0.083)	(0.092)	(0.086)	(0.252)
ln(area)	−0.646***	−0.751***	−0.689***	−0.600***
	(0.100)	(0.101)	(0.093)	(0.161)
Cowpea				
CA (= 1)	−0.742	−0.929	−1.188	1.829
	(1.141)	(1.415)	(1.439)	(3.818)
rainfall shortage	−0.628		−1.493***	−2.468**
	(0.477)		(0.553)	(1.221)
CA × rainfall shortage	3.171***		3.763***	6.006**
	(1.008)		(1.272)	(2.558)
rainfall surplus		−1.103**	−1.492***	−0.970
		(0.482)	(0.557)	(1.002)
CA × rainfall surplus		−0.286	1.234	3.148
		(1.309)	(1.624)	(2.543)
ln(basal)	0.073	0.168	0.090	−0.162
	(0.098)	(0.104)	(0.098)	(0.276)
ln(top)	0.003	0.092	−0.008	−0.650
	(0.205)	(0.218)	(0.207)	(0.569)
ln(seed)	0.451***	0.454***	0.463***	0.659**
	(0.168)	(0.171)	(0.170)	(0.287)
ln(area)	−0.720***	−0.799***	−0.723***	−0.491*
	(0.162)	(0.169)	(0.162)	(0.291)
Type of Effect	Household FE	Household FE	Household FE	Plot MCD
Observations	7,643	7,643	7,643	5,004
Log Likelihood	-15,908	-16,406	-15,902	-12,268
Kleibergen-Paap LM stat	22.96***	23.02***	25.23***	3.790**
Anderson-Rubin Wald stat	22.10**	29.68***	44.79***	38.53***
Kleibergen-Paap Wald stat	1.930*	1.989*	1.444	0.251

Note: Dependent variable is log of yield. All specifications include crop-specific intercept terms and year dummies. In each regression the adoption of CA is treated as endogenous and is instrumented with the Inverse Mills Ratio (IMR) calculated from the predicted values of zero-stage probits which are presented in the Appendix, Table B3. The CA × rainfall shortage and CA × rainfall surplus terms are also treated as endogenous and instrumented using the interaction of the IMR and the rainfall terms. The null hypothesis of the Kleibergen-Paap LM test is that the rank condition fails (i.e., the first-stage equation is underidentified). The null hypothesis of the Anderson-Rubin Wald test is that the coefficients on the endogenous regressors in the structural equation are jointly equal to zero (i.e., the instruments in the first-stage equation are weak). The null hypothesis of the Kleibergen-Paap Wald test is that a t-test at the 5% significance level on the coefficients of the endogenous regressors rejects no more than 25% of the time (i.e., the instruments in the first-stage equation are weak). Standard errors clustered by household and crop are reported in parentheses (*p<0.1; **p<0.05; ***p<0.01).

Table C1: Zero-Stage Tobit with Rain Shortage and Surplus

	(1)	(2)
Number of HH in ward with NGO support	0.005** (0.002)	0.007*** (0.001)
Household Control	FE	MCD
Observations	7,643	7,643
Log Likelihood	-4,310	-4,870

Note: Dependent variable is an indicator for whether or not CA was used on the plot. Though not reported, all Tobit regressions include crop-specific inputs and intercept terms, and year dummies. Column (1) includes household fixed effects. Column (2) includes the Mundlak-Chamberlain device. Standard errors clustered by household and crop are reported in parentheses (*p<0.1; **p<0.05; ***p<0.01).

Table C2: Yield Function using Zero-Stage Tobit

	(1)	(2)
Maize		
CA (= 1)	−0.141	−0.611
	(0.448)	(0.736)
rainfall shortage	−0.366	−0.639
	(0.365)	(0.440)
CA × rainfall shortage	0.216	0.958
	(0.684)	(0.927)
rainfall surplus	−1.329***	−1.659***
	(0.313)	(0.321)
CA × rainfall surplus	1.544***	2.450***
	(0.533)	(0.613)
Sorghum		
CA (= 1)	−0.185	−0.099
	(0.763)	(1.002)
rainfall shortage	−1.770***	−1.929***
	(0.417)	(0.434)
CA × rainfall shortage	2.460**	3.335***
	(1.105)	(1.243)
rainfall surplus	−1.207***	−1.410***
	(0.365)	(0.364)
CA × rainfall surplus	0.579	1.141
	(0.787)	(0.838)
Millet		
CA (= 1)	−1.942	−1.352
	(1.749)	(1.767)
rainfall shortage	−1.859***	−1.866***
	(0.629)	(0.637)
CA × rainfall shortage	3.248	3.356
	(2.573)	(2.629)
rainfall surplus	−1.595***	−1.699***
	(0.308)	(0.299)
CA × rainfall surplus	2.778	3.663*
	(1.819)	(2.030)
Groundnut		
CA (= 1)	0.177	0.333
	(0.535)	(0.627)
rainfall shortage	−1.258***	−1.368***
	(0.318)	(0.321)
CA × rainfall shortage	0.555	1.049
	(0.731)	(0.808)
rainfall surplus	−0.061	−0.088
	(0.248)	(0.246)
CA × rainfall surplus	−0.321	−0.339
	(0.601)	(0.673)
Cowpea		
CA (= 1)	−0.800	−0.606
	(0.734)	(0.992)
rainfall shortage	−1.430***	−1.500***
	(0.533)	(0.550)
CA × rainfall shortage	3.558***	3.802***
	(1.027)	(1.189)
rainfall surplus	−1.572***	−1.440***
	(0.536)	(0.537)
CA × rainfall surplus	1.733	1.234
	(1.421)	(1.604)
Household FE	Yes	Yes
Observations	7,643	7,643
Kleibergen-Paap LM Stat	279.51***	182.71***
Log Likelihood	-15,737	-15,770

Note: Dependent variable is log of yield. Though not reported, all specifications include crop-specific inputs and intercept terms, and year dummies. Column (1) uses the predicted value and the Inverse Mills Ratio from the zero-stage Tobit with household fixed effects as presented in column (1) of Table C1. Column (2) uses the predicted value and the Inverse Mills Ratio from the zero-stage Tobit with household a Mundlak-Chamberlain device as presented in column (2) of Table C1. All standard errors are clustered by household and crop and are reported in parentheses (*p<0.1; **p<0.05; ***p<0.01).

Table C3: Placebo Tests

	Future Adopter v. Conventional (1)	Future Disadopter v. Conventional (2)
Maize		
CA (= 1)	0.326	0.093
	(0.606)	(0.293)
rainfall shock	−0.333	−0.492*
	(0.290)	(0.283)
CA × rainfall shock	−0.924	−0.611
	(0.921)	(0.648)
Sorghum		
CA (= 1)	1.329**	0.222
	(0.530)	(0.464)
rainfall shock	−1.063***	−1.292***
	(0.390)	(0.387)
CA × rainfall shock	−1.419*	0.391
	(0.809)	(0.865)
Millet		
CA (= 1)	−0.928	0.063
	(0.772)	(0.382)
rainfall shock	−1.188***	−1.379***
	(0.415)	(0.405)
CA × rainfall shock	0.555	0.000
	(1.270)	(0.588)
Groundnut		
CA (= 1)	0.048	0.370
	(0.710)	(0.257)
rainfall shock	−0.200	−0.206
	(0.287)	(0.284)
CA × rainfall shock	−0.423	−0.225
	(1.125)	(0.589)
Cowpea		
CA (= 1)	0.052	0.264
	(1.094)	(0.849)
rainfall shock	−1.116**	−1.274**
	(0.553)	(0.543)
CA × rainfall shock	0.296	0.106
	(1.432)	(1.862)
Observations	4,132	4,629
R^2	0.928	0.924

Note: Dependent variable is log of yield. Though not reported, all specifications include crop-specific inputs and intercept terms, and year dummies. Column (1) uses a pseduo-treatment in which "treated" plots are those that will, sometime in the future (2009, 10, or 11), have CA but at the moment are cultivated using conventional methods. These are compared to "control" plots which never have CA and are always cultivated using conventional methods. Column (2) uses a pseduo-treatment in which "treated" plots are those that were, sometime in the past (2008, 09, or 10), CA plots but at the moment are cultivated using conventional methods. These are compared to "control" plots which never have CA and are always cultivated using conventional methods. All standard errors are clustered by household and crop and are reported in parentheses (*p<0.1; **p<0.05; ***p<0.01).

Table D1: Yield Function Using Three out of Four Years

	All Years (1)	W/O 2011 (2)	W/O 2010 (3)	W/O 2009 (4)	W/O 2008 (5)
Maize					
CA (= 1)	−2.853**	4.907	−3.570***	−2.764**	−5.010***
	(1.141)	(3.036)	(1.195)	(1.169)	(1.242)
rainfall shock	−1.675***	−0.382	−1.493***	−1.645***	−2.041***
	(0.353)	(0.691)	(0.376)	(0.395)	(0.456)
CA × rainfall shock	2.628***	−0.255	2.679***	2.722***	3.316***
	(0.704)	(1.271)	(0.670)	(0.809)	(1.082)
Sorghum					
CA (= 1)	−0.171	−0.592	−0.571	0.134	−0.543
	(1.603)	(2.709)	(1.854)	(1.513)	(1.914)
rainfall shock	−1.476***	−1.118**	−1.082**	−1.555***	−1.323***
	(0.353)	(0.466)	(0.440)	(0.505)	(0.386)
CA × rainfall shock	0.899	0.800	0.597	0.937	0.508
	(0.904)	(1.284)	(0.932)	(1.293)	(1.092)
Millet					
CA (= 1)	−3.784	−3.881	−2.160	0.308	−10.450
	(3.808)	(3.431)	(2.638)	(4.022)	(9.108)
rainfall shock	−1.715***	−1.605***	−0.102	−1.418***	−2.234***
	(0.341)	(0.442)	(0.459)	(0.448)	(0.398)
CA × rainfall shock	3.357	3.810*	−4.656	3.092	5.284
	(2.279)	(2.256)	(5.458)	(3.555)	(6.466)
Groundnut					
CA (= 1)	−0.272	−0.947	−1.726	2.370	−3.185**
	(1.324)	(1.526)	(1.494)	(1.603)	(1.597)
rainfall shock	−0.950***	−0.864***	−0.756**	−1.095***	−0.865***
	(0.265)	(0.287)	(0.362)	(0.306)	(0.326)
CA × rainfall shock	1.433*	1.498**	2.633***	0.245	1.443
	(0.832)	(0.759)	(0.961)	(1.058)	(1.296)
Cowpea					
CA (= 1)	−1.564	−0.401	−2.818*	−1.022	−2.622*
	(1.349)	(1.698)	(1.624)	(1.482)	(1.479)
rainfall shock	−1.606***	−1.438**	−1.410**	−1.666***	−1.274**
	(0.485)	(0.604)	(0.639)	(0.547)	(0.553)
CA × rainfall shock	2.123	1.690	2.719*	1.783	1.887
	(1.404)	(1.681)	(1.652)	(1.604)	(1.582)
Household FE	Yes	Yes	Yes	Yes	Yes
Observations	7,643	5,300	5,527	5,911	6,191
Kleibergen-Paap LM Stat	33.8***	11.0***	19.4***	61.7***	16.8***
Log Likelihood	-16,192	-11,684	-11,478	-12,809	-13,624

Note: For purposes of comparison, column (1) reproduces our preferred results from column (4) in Table 7 in the paper. Columns (2) - (5) use the same specification as in column (1) but in each case we remove one year of the data. Standard errors are clustered by household and crop (*p<0.1; **p<0.05; ***p<0.01).

Table D2: Yield Function Using Three out of Four Years with Rain Shortage and Surplus

	All Years (1)	W/O 2011 (2)	W/O 2010 (3)	W/O 2009 (4)	W/O 2008 (5)
Maize					
CA (= 1)	−1.893	6.615**	−4.551**	−0.502	−4.133***
	(1.472)	(3.092)	(1.876)	(1.545)	(1.359)
rainfall shortage	−1.076**	2.821**	−2.021***	−0.063	−1.605***
	(0.481)	(1.357)	(0.481)	(0.562)	(0.502)
CA × rainfall shortage	2.098**	−6.690***	1.512	3.436***	3.345***
	(0.987)	(2.459)	(1.068)	(1.209)	(1.153)
rainfall surplus	−1.884***	0.097	−1.066**	−2.103***	−2.443***
	(0.358)	(0.719)	(0.472)	(0.434)	(0.468)
CA × rainfall surplus	2.931***	−1.161	2.070***	3.101***	3.382***
	(0.657)	(1.324)	(0.745)	(0.754)	(1.032)
Sorghum					
CA (= 1)	−0.512	0.196	−1.031	−1.548	−0.871
	(1.780)	(2.644)	(2.707)	(1.919)	(1.900)
rainfall shortage	−1.702***	−0.783	−1.273**	−1.232*	−1.570***
	(0.454)	(0.682)	(0.512)	(0.677)	(0.508)
CA × rainfall shortage	2.356*	2.644	−0.379	−0.374	1.763
	(1.326)	(1.615)	(1.912)	(2.418)	(1.417)
rainfall surplus	−1.214***	−1.042**	−1.480***	−1.100*	−0.823**
	(0.377)	(0.462)	(0.543)	(0.564)	(0.411)
CA × rainfall surplus	0.464	0.346	1.375	0.581	−0.300
	(0.987)	(1.300)	(0.969)	(1.365)	(1.177)
Millet					
CA (= 1)	−2.671	−2.484	−0.528	2.747	−8.180
	(3.293)	(3.601)	(2.593)	(3.679)	(8.628)
rainfall shortage	−1.604**	−1.857	0.442	−0.624	−1.938***
	(0.658)	(1.220)	(0.985)	(0.692)	(0.659)
CA × rainfall shortage	1.930	3.886	−8.037	7.960	6.055
	(2.344)	(3.689)	(7.069)	(7.937)	(7.729)
rainfall surplus	−1.689***	−1.426***	−0.305	−1.670***	−2.146***
	(0.320)	(0.433)	(0.433)	(0.531)	(0.365)
CA × rainfall surplus	3.170	3.131	−3.628	1.877	2.374
	(2.202)	(2.151)	(5.522)	(4.286)	(4.797)
Groundnut					
CA (= 1)	−0.266	0.313	−2.366	2.376	−2.989*
	(1.499)	(1.634)	(2.214)	(2.263)	(1.626)
rainfall shortage	−1.229***	−2.880***	−1.211***	−0.471	−0.971**
	(0.338)	(0.777)	(0.406)	(0.404)	(0.385)
CA × rainfall shortage	0.405	3.512***	0.407	2.219	1.814
	(0.984)	(1.338)	(1.104)	(2.781)	(1.373)
rainfall surplus	−0.170	−0.930***	−0.388	−0.511	−0.121
	(0.281)	(0.299)	(0.571)	(0.316)	(0.339)
CA × rainfall surplus	−0.205	1.182	2.342*	−1.186	−2.554
	(0.985)	(0.800)	(1.384)	(1.261)	(1.994)
Cowpea					
CA (= 1)	−1.188	−0.171	−3.095	−0.963	−2.171
	(1.439)	(1.715)	(2.233)	(1.661)	(1.503)
rainfall shortage	−1.493***	−1.195	−1.182	−0.850	−1.219**
	(0.553)	(0.848)	(0.756)	(0.670)	(0.591)
CA × rainfall shortage	3.763***	4.783***	3.580**	1.827	3.214**
	(1.272)	(1.743)	(1.640)	(1.684)	(1.401)
rainfall surplus	−1.492***	−1.418**	−1.835**	−1.610**	−0.697
	(0.557)	(0.613)	(0.814)	(0.635)	(0.695)
CA × rainfall surplus	1.234	1.241	3.860*	1.656	−1.581
	(1.624)	(1.744)	(2.112)	(1.825)	(2.609)
Household FE	Yes	Yes	Yes	Yes	Yes
Observations	7,643	5,300	5,527	5,911	6,191
Kleibergen-Paap LM Stat	25.23***	11.35***	9.05***	37.3***	18.2***
Log Likelihood	-15,902	-11,967	-11,949	-12,561	-13,245

Note: For purposes of comparison, column (1) reproduces our preferred results from column (3) in Table 8 in the paper. Columns (2) - (5) use the same specification as in column (1) but in each case we remove one year of the data. Standard errors are clustered by household and crop (*p<0.1; **p<0.05; ***p<0.01).

Table D3: Yield Function with Alternative Rainfall Measures

	(1)	(2)
Maize		
CA (= 1)	−2.411**	−3.587***
	(1.130)	(1.240)
rainfall shock	−1.050***	−0.879***
	(0.309)	(0.279)
CA × rainfall shock	2.095***	2.153***
	(0.546)	(0.544)
Sorghum		
CA (= 1)	−0.538	−1.511
	(1.480)	(1.628)
rainfall shock	−0.735**	−0.753***
	(0.286)	(0.286)
CA × rainfall shock	0.874	0.546
	(0.601)	(0.605)
Millet		
CA (= 1)	−3.088	−4.552
	(2.841)	(3.115)
rainfall shock	−0.602*	−0.764**
	(0.311)	(0.316)
CA × rainfall shock	−1.687	−1.063
	(2.346)	(1.663)
Groundnut		
CA (= 1)	−0.178	−1.283
	(1.194)	(1.270)
rainfall shock	−0.498**	−0.487**
	(0.245)	(0.219)
CA × rainfall shock	1.095*	1.276**
	(0.631)	(0.608)
Cowpea		
CA (= 1)	−1.024	−1.969
	(1.145)	(1.281)
rainfall shock	−0.798*	−1.207***
	(0.433)	(0.419)
CA × rainfall shock	0.411	1.847*
	(1.339)	(1.094)
Household FE	Yes	Yes
Kleibergen-Paap LM	30.05***	26.68***
Observations	7,643	76,43
Log Likelihood	-16,417	-16,800

Note: Dependent variable is log of yield. All specifications include crop-specific intercept terms and year dummies. In each regression the adoption of CA is treated as endogenous and is instrumented as previously discussed. Column (1) replaces any deviation in rainfall that is with ± one standard deviation with a zero. Thus, any realized value that is $0.202 \leq R_{wt} \leq 0.749$ is set to zero. Column (2) replaces any deviation in rainfall that is with ± one half of a standard deviation with a zero. Thus, any realized value that is $0.339 \leq R_{wt} \leq 0.612$ is set to zero. Standard errors clustered by household and crop are reported in parentheses (*p<0.1; **p<0.05; ***p<0.01).
